# Conley-Morse-Forman theory for generalized combinatorial multivector fields on finite topological spaces

**DOI:** 10.1007/s41468-022-00102-9

**Published:** 2022-10-05

**Authors:** Michał Lipiński, Jacek Kubica, Marian Mrozek, Thomas Wanner

**Affiliations:** 1 Division of Computational Mathematics, Faculty of Mathematics and Computer Science, Jagiellonian University, ul. St. Łojasiewicza 6, 30-348 Kraków, Poland; 2 Department of Mathematical Sciences, George Mason University, Fairfax, VA 22030 USA

**Keywords:** Combinatorial vector field, Finite topological space, Discrete Morse theory, Isolated invariant set, Conley theory, Primary 37B30, Secondary 37E15, 57M99, 57Q05, 57Q15

## Abstract

We generalize and extend the Conley-Morse-Forman theory for combinatorial multivector fields introduced in Mrozek (Found Comput Math 17(6):1585–1633, 2017). The generalization is threefold. First, we drop the restraining assumption in Mrozek (Found Comput Math 17(6):1585–1633, 2017) that every multivector must have a unique maximal element. Second, we define the dynamical system induced by the multivector field in a less restrictive way. Finally, we also change the setting from Lefschetz complexes to finite topological spaces. Formally, the new setting is more general, because every Lefschetz complex is a finite topological space, but the main reason for switching to finite topologcial spaces is because the latter better explain some peculiarities of combinatorial topological dynamics. We define isolated invariant sets, isolating neighborhoods, Conley index and Morse decompositions. We also establish the additivity property of the Conley index and the Morse inequalities.

## Introduction

The combinatorial approach to dynamics has its origins in two papers by Robin Forman (Forman 1998a, b) published in the late 1990s. Central to the work of Forman is the concept of a combinatorial vector field. One can think of a combinatorial vector field as a partition of the collection of cells of a cellular complex into combinatorial vectors which may be singletons (critical vectors or critical cells) or doubletons such that one element of the doubleton is a face of codimension one of the other (regular vectors). The original motivation of Forman was the presentation of a combinatorial analogue of classical Morse theory. However, soon the potential for applications of such an approach was discovered in data science. Namely, the concept of combinatorial vector field enables direct applications of the ideas of topological dynamics to data and eliminates the need of the cumbersome construction of a classical vector field from data.

Recently, Batko et al. (2020), Kaczynski et al. (2016), Mrozek and Wanner (2021), in an attempt to build formal ties between the classical and combinatorial Morse theory, extended the combinatorial theory of Forman to Conley theory (Conley 1978), a generalization of Morse theory. In particular, they defined the concept of an isolated invariant set, the Conley index and Morse decomposition in the case of a combinatorial vector field on the collection of simplices of a simplicial complex. Later, Mrozek (2017) observed that certain dynamical structures, in particular homoclinic connections, cannot have an analogue for combinatorial vector fields and as a remedy proposed an extension of the concept of combinatorial vector field, a *combinatorial multivector field*. We recall that in the collection of cells of a cellular complex there is a natural partial order induced by the face relation. Every combinatorial vector in the sense of Forman is convex with respect to this partial order. A combinatorial multivector in the sense of Mrozek (2017) is defined as a convex collection of cells with a unique maximal element, and a combinatorial multivector field is then defined as a partition of cells into multivectors. The results of Mrozek (2017) were presented in the algebraic setting of chain complexes with a distinguished basis (Lefschetz complexes), an abstraction of the chain complex of a cellular complex already studied by Lefschetz (1942). The results of Forman were earlier generalized to the setting of Lefschetz complexes in Jöllenbeck and Welker (2009); Kozlov (2005); Sköldberg (2005), and to the more general setting of finite topological spaces in Minian (2012). Note that the setting of finite topological spaces is more general, because every Lefschetz complex is a poset via the face relation, therefore also a finite topological space via the Alexandrov Theorem (Alexandrov 1937).

The aim of this paper is a threefold advancement of the results of Mrozek (2017). We generalize the concept of combinatorial multivector field by lifting the assumption that a multivector has a unique maximal element. This assumption was introduced in Mrozek (2017) for technical reasons, but turned out to be a barrier for adapting the techniques of continuation in topological dynamics to the combinatorial setting (Dey et al. 2022). We define the dynamics associated with a combinatorial multivector field following the ideas of Dey et al. (2019). This approach is less restrictive, and better adjusted to persistence of Conley index (Dey et al. 2022, 2020, 2022). Finally, we change the setting from Lefschetz complexes to finite topological spaces. Here, the generalization is not the main motivation. We do so, because the specific nature of finite topological spaces helps explain the differences between the combinatorial and the classical theory. For instance, isolated invariant sets are always closed in the classical theory, but this is not true in its combinatorial counterpart, because separation in finite topological spaces is only $T_0$.

In this extended and generalized setting we define the concepts of isolated invariant set and Conley index. We also define attractors, repellers, attractor-repeller pairs and Morse decompositions, and provide a topological characterization of attractors and repellers. Furthermore, we prove the Morse equation for Morse decompositions, and finally deduce from it the Morse inequalities.

We note that, as in the classical case, attractors of multivector fields form a bounded, distributive lattice. Therefore, the algebraic characterization of lattices of attractors developed by Kalies, Mischaikow, and Vandervorst in Kalies et al. (2014, 2016, 2021) applies also to the combinatorial case. What unites the two approaches is the combinatorial multivalued map. The difference is that the approach in Kalies et al. (2014, 2016, 2021) is purely algebraic whereas our approach is purely topological. The relation between the algebraic and topological approaches in the case of gradient-like dynamics is very interesting, but beyond the scope of the present paper. We leave the study of this relation for future investigation.

The organization of the paper is as follows. In Sect. 2 we present the main results of the paper. This is an informal section aiming at the presentation of the motivation, intuition, and main ideas of the paper on the basis of an elementary geometric example. The reader interested only in the formal results and their correctness may skip this section. In Sect. 3 we recall basic concepts and facts needed in the paper. Section 4 is devoted to the study of the dynamics of combinatorial multivector fields and the introduction of isolated invariant sets. In Sect. 5 we define index pairs and the Conley index. In Sect. 6 we investigate limit sets, attractors and repellers in the combinatorial setting. Finally, Sect. 7 is concerned with Morse decompositions and Morse inequalities for combinatorial multivector fields.

## Main results

In this section we present the main results of the paper in an informal and intuitive way, for a simple simplicial example. We also indicate the main conceptual differences between our combinatorial approach and the classical theory. Precise definitions and statements are given in the following sections.

### Combinatorial phase space

In this paper we study dynamics in finite spaces. We also refer to finite spaces as combinatorial spaces. In applications, they typically are collections of cells of a simplicial, a cubical, or a more general cellular complex. All such collections are examples of Lefschetz complexes. We recall that a *Lefschetz complex* (see Sect. 3.5 for precise definitions) is a distinguished basis of a finitely generated chain complex. By *Lefschetz homology* we mean the homology of this chain complex. In a Lefschetz complex, as in every cellular complex, there is a well defined face relation which makes every Lefschetz complex a finite poset and, via the Alexandrov Theorem (Alexandrov 1937), a finite topological space. The results of this paper apply to any finite topological space, although from the point of view of applications Lefschetz complexes remain the main object of interest. However, the viewpoint from the perspective of finite topological spaces is closer to geometric intuition and, as pointed out in the introduction, helps explain similarities and differences between the classical and the combinatorial theory.

A Lefschetz complex, as every topological space, has also well defined singular homology. Note that the singular homology and Lefschetz homology of a Lefschetz complex need not be the same in general, although they are the same for many concrete examples of Lefschetz complexes, in particular for cellular complexes. The results of this paper apply to any homology theory for which the excision and Mayer-Vietoris theorems hold. In particular, they apply to singular homology of finite topological spaces and Lefschetz homology of Lefschetz complexes regardless whether they are the same or not. But, they depend on the specifically chosen homology theory.

**Fig. 1:**
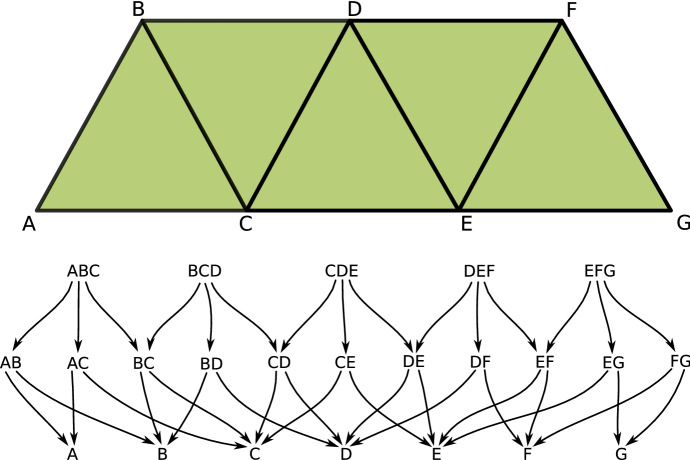
An example of a simplicial complex (top) and the poset (a finite $T_0$ topological space, bottom) induced by its face relation

For concrete Lefschetz complexes such as simplicial complexes there is also topology of its geometric realization which is very different from the finite topology of a Lefschetz complex. Nevertheless, via McCord’s Theorem (McCord 1966), these topologies are weakly homotopic and, consequently, their algebraic invariants such as homotopy and singular homology groups are the same. As an example consider the family *X* of all simplices of the simplicial complex in Fig. 1 (top). The associated face poset which makes *X* a finite topological space is presented in Fig. 1 (bottom). Another topological space associated with the simplicial complex is its *polytope*, that is the union of all its simplexes with topology induced from the plane. Although the two topological spaces are clearly very different, in particular the poset is only $T_0$ and the polytope is $T_2$, they are related. We can see it by identifying the simplices in the poset with open simplices in the polytope. In this case, the set $A\subset X$ is open (respectively closed) in the $T_0$ topology of *X* if and only if the union of the corresponding open simplices is open (respectively closed) in the Hausdorff topology of the polytope of *X*.

**Fig. 2:**
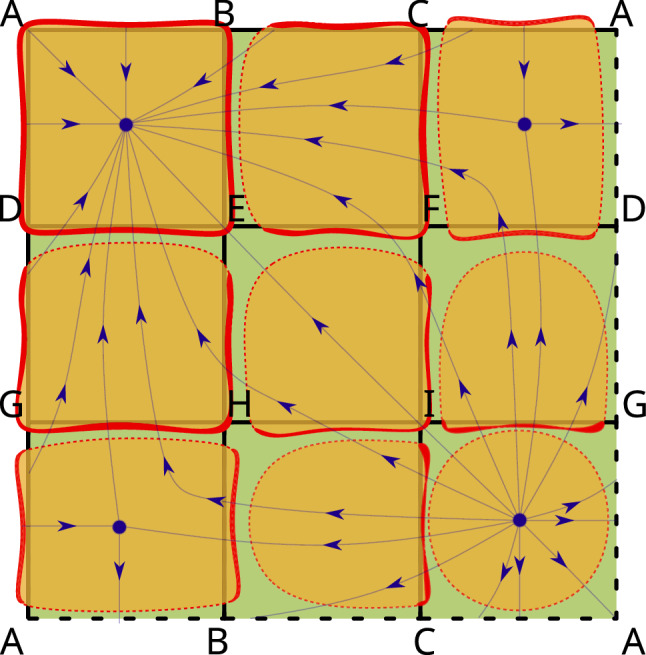
A flow on a torus *T* represented as a green square with bottom and top as well as left and right faces identified. A cellular structure $\mathcal {T}$ is imposed on *T*. It consists of 9 squares, 18 edges and 9 vertices. The flowlines cross the edges transversally. This leads to a multivector field on $\mathcal {T}$. Each multivector consists of cells intersected by the same orange region. For instance, consider the top right orange region. It represents a multivector consisting of three cells: square *CADF* and edges *CA*,*DF*, because these are the only cells in the boundary of *CADF* crossed by flowlines towards *CADF*. Similarly, the orange region in bottom right consists only of square *ACIG*, because none of its faces is crossed inwards

### Combinatorial dynamical systems

Classical dynamical systems, when considered on a finite space, are very restrictive. On the one hand, as observed in [Chocano et al. 2021, Theorem 2.6], a flow on a finite $T_0$ topological space necessarily has only stationary trajectories. On the other hand, it is straightforward to check that in the same setting but for a dynamical system with discrete time every trajectory is periodic. Hence, to allow for more interesting dynamics in finite spaces, we consider *combinatorial dynamical systems* by which we mean iterates of a multivalued map acting on the finite space (see Sect. 4.1 for precise definitions). The question remains whether one can still distinguish a class of multivalued maps whose dynamics is flow-like. One would expect that for a general multivalued map trajectories may arbitrarily jump through space, whereas in the case of flow-like dynamics they should join neighbors in the topological space. We do not attempt to formalize the flow-like concept. Instead, inspired by Forman (Forman 1998a, b), we study the dynamics of a special class of multivalued maps on finite spaces, generated by a combinatorial analogue of a vector field. We introduce it in the next section. Clearly, the dynamics of general multivalued maps in finite spaces, corresponding to classical discrete time dynamics, is also of interest. However, Conley theory for general multivalued maps in finite topological spaces requires different ideas and is beyond the scope of the present paper and will be presented in [3]

### Combinatorial multivector fields

A *combinatorial multivector field* (see Sect. 4.3 for precise definitions and more details) is a partition of a finite topological space *X* into *locally closed sets* (or *convex* sets in terms of posets, see Proposition 3.10), that is, sets $A\subset X$ such that their *mouth* ${\text {mo}}A:={\text {cl}}A\setminus A$ (the closure of *A* with *A* removed) is closed. The elements of the partition are referred to as *multivectors*.

There are many ways to obtain multivector fields in applications. One of the most intuitive methods is via the transversal polygons construction. It consists in the approximation of a flow on a manifold by the decomposition of the manifold into convex polygonal cells in such a way that the flow lines cross the faces of every cell transversally. The importance of such decompositions was indicated already by Boczko et al. (2007). The top dimensional cells together with their faces (lower dimensional cells) form a cellular decomposition of the manifold. The transversality and the convexity then imply that flowlines originating in the same face enter the same top dimensional cell. By grouping each top dimensional cell with all its faces entering the cell, one obtains a combinatorial multivector field (see Mrozek et al. (2022) for details). An example of such a construction is presented in Fig. 2. A special feature of a multivector field constructed this way is that every multivector contains a cell of maximal dimension and is contained in the closure of the cell. This need not be the case in general as the following example shows.

#### Example 2.1

**Fig. 3:**
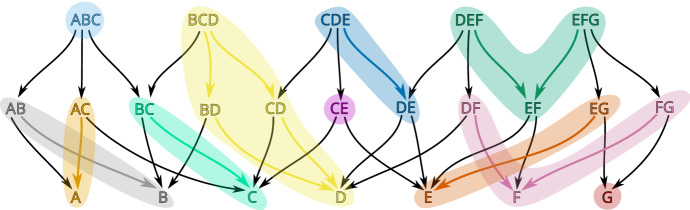
A partition of a poset into multivectors (convex subsets). Nodes as well as corresponding arrows of each multivector are highlighted with a distinct color

**Fig. 4:**
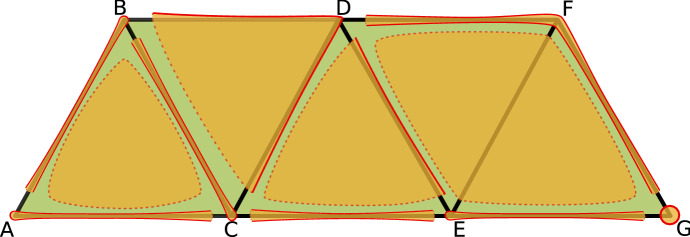
A geometric visualization of the combinatorial multivector field in Fig. 3. A multivector may be considered as a “black box” whose dynamics is known only via splitting its boundary into the exit and entrance parts

Consider the partition

$$\begin{aligned} \mathcal {V}:=\{\;\{A, AC\}, \{ABC\}, \{B, AB\}, \{C, BC\}, \{CE\}, \{D, BD, CD, BCD\},\\ \{DE, CDE\}, \{E, EG\}, \{EF, DEF, EFG\}, \{F, DF, FG\}, \{G\}\; \} \end{aligned}$$

of the set of cells *X* of the simplicial complex in Fig. 1. It is easy to check that this partition is a multivector field presented in Fig. 3 with *X* visualized as a poset, and in Fig. 4 with *X* visualized as a simplicial complex. Every multivector in Fig. 3 is highlighted with a different color and in Fig. 4 it is indicated by an orange region as in Fig. 2. In terms of the transversal polygons interpretation the dotted part of the boundary of a multivector indicates the outward-directed flow while the solid part of the boundary indicates the inward flow.

As this example indicates, a multivector may contain no top dimensional cell but also more than one top dimensional cell. Such multivectors, in particular, are useful in constructing multivector fields from clouds of vectors.

Note that in the case of a multivector field constructed from transversal polygons, the transversality implies that the flow may exit the closure of a multivector *V* only through its mouth. Hence, ${\text {cl}}V$ may be interpreted as an isolating block for the flow with exit set ${\text {mo}}V$. This allows us to think of a multivector as a black box where the dynamics is known only at its boundary, but not inside. Moreover, the relative homology $H({\text {cl}}V, {\text {mo}}V)$ may be interpreted as the Conley index of the invariant set of the flow isolated by ${\text {cl}}V$ (for the definition of Conley index and isolating block in the classical setting see Conley 1978; Conley and Easton 1971; Stephens and Wanner 2014).

### Combinatorial flow associated with a multivector field

With every combinatorial multivector field $\mathcal {V}$ on a finite topological space *X* we associate a combinatorial dynamical system induced by the multivalued map $\Pi _\mathcal {V}:X\multimap X$ given by

1 $$\begin{aligned} \Pi _\mathcal {V}(x):= {\text {cl}}x \cup [x]_\mathcal {V}, \end{aligned}$$

where $[x]_\mathcal {V}$ denotes the unique multivector in $\mathcal {V}$ containing *x*. Similarly to Forman (1998a) we often refer to the combinatorial dynamical system given by (1) as the *combinatorial flow* associated with the multivector field $\mathcal {V}$.

Formula (1) says that starting from cell *x* we can either go to the closure of *x* or we can stay in the multivector of *x*. In the case of a multivector field constructed from transversal polygons as in Fig. 2 the first case may be interpreted as the flow-like behaviour, because a flow line cannot leave cell *x* without crossing the boundary of *x*. The second case reflects the black box nature of a multivector: we only know what happens at the boundary of a multivector, therefore we do not want to exclude any movement inside a multivector.

### Graph interpretation

**Fig. 5:**
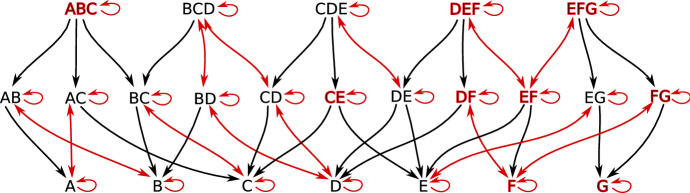
The combinatorial flow $\Pi _\mathcal {V}$ of the multivector field in Figs. 3 and 4 represented as the digraph $G_\mathcal {V}$. Downward arrows are induced by the closure components of $\Pi _\mathcal {V}$. Bi-directional edges and self-loops reflect dynamics within multivectors. For clarity, we omit edges that can be obtained by between-level transitivity, e.g., the bi-directional connection between node *D* and *BCD*. The nodes of critical multivectors are bolded in red

Let $F:X\multimap X$ be an arbitrary multivalued map acting on a finite topological space *X*. The combinatorial dynamical system induced by *F*, as in Kalies et al. (2016), may be interpreted as a directed graph $G_F$ whose vertices are the elements of *X* and there is a directed arrow from *x* to *y* whenever $y\in F(x)$. The graph $G_\mathcal {V}:=G_{\Pi _\mathcal {V}}$ of the combinatorial flow discussed in Example 2.1 is presented in Fig. 5.

A basic concept of multivalued dynamics, a *solution*, corresponds to a walk in $G_F$. We are interested in *full solutions*, that is, bi-infinite walks, as well as *paths* by which we mean finite walks (see Sect. 4.2 for precise definitions and more details).

Translation of problems in combinatorial topological dynamics to the language of directed graphs facilitates their algorithmic study. However, we emphasize that combinatorial topological dynamics cannot be reduced just to graph theory, because the topology in the set of vertices of the directed graph matters, as we will see in the following sections, in particular in the concept of essential solution introduced in the next section. In consequence, combinatorial topological dynamics as a field is a part of general topological dynamics and not a part of graph theory. The use of the classical terminology of dynamics also in the combinatorial setting helps focusing on this difference.

### Essential solutions

As we already explained, formula (1) for the combinatorial dynamical system associated with a combinatorial multivector field has a natural geometric interpretation. However, it also has a drawback, because, as one can easily check, for such a combinatorial dynamical system there is a stationary (constant) solution through each point. This, in particular, is the consequence of the black box nature of a multivector and the tightness of a finite topological space. We overcome this problem by distinguishing regular and critical mulltivectors. To define them we fix a homology theory in the finite topological space *X*. For examples based on simplicial complexes we just take simplicial homology which in this case coincides with Lefschetz homology and singular homology (see Sect. 3.6).

We say that a multivector *V* is *critical* if $H({\text {cl}}V, {\text {mo}}V)\ne 0$. Otherwise we call *V* *regular*. There are five critical multivectors in the multivector field presented in Example 2.1:

$$\begin{aligned} \{ABC\},\ \{CE\},\ \{DEF,EF,EFG\},\ \{DF,F,FG\},\ \{G\}. \end{aligned}$$

In terms of the transversal polygon interpetation a critical multivector may be considered as an isolating block with a non-trivial Conley index. Therefore, accepting the existence of a non-empty invariant set inside may be justified by the Ważewski property of the Conley index. In the case of a regular multivector we have no topological justification to expect a non-empty invariant set. This motivates the introduction of essential solutions. An *essential solution* is a full solution $\gamma$ such that if $\gamma (t)$ belongs to a regular multivector $V\in \mathcal {V}$ then there exist both a $k>0$ and an $l<0$ satisfying the exclusions $\gamma (t+k),\gamma (t+l)\not \in V$ (see Sect. 4.4 for precise definition and more details). An example of an essential solution $\gamma :{\mathbb {Z}}\rightarrow X$ for the multivector field in Fig. 4 is given by:

$$\begin{aligned} \gamma (t) = {\left\{ \begin{array}{ll} CE &{} t < 0,\\ E &{} t \in \{0,2,3\},\\ EG &{} t \in \{1, 4\},\\ G &{} t > 4. \end{array}\right. } \end{aligned}$$

### Isolated invariant sets and Conley index

We say that a set $S\subset X$ is *invariant* if every $x\in S$ admits an essential solution through *x* in *S*. We say that an invariant set *S* is an *isolated invariant* set if there exists a closed set *N*, called an *isolating set* such that $S\subset N$, $\Pi _\mathcal {V}(S)\subset N$, and every path in *N* with endpoints in *S* is a path in *S* (see Sect. 4.5 for precise definitions and more details). Note that our concept of isolating set is weaker than the classical concept of isolating neighborhood, because the maximal invariant subset of *N* may not be contained in the interior of *N*. The need of a weaker concept is motivated by the tightness in finite topological spaces. In particular, an isolated invariant set *S* may intersect the closure of another isolated invariant set $S'$ and be disjoint but not disconnected from $S'$. For instance, with respect to Example 2.1 the sets $S_1:=\{A, AC, C, BC, B, AB\}$ and $S_2:=\{ABC\}$ are both isolated invariant sets isolated respectively by $N_1:=S_1$ and $N_2:={\text {cl}}S_1 = S_1\cup S_2$. Observe that $S_1\subset N_2$. Thus, the isolating set in the combinatorial setting of finite topological spaces is a relative concept. Therefore, one has to specify each time which invariant set is considered as being isolated by a given isolating set.

Given an isolated invariant set *S* of a combinatorial multivector field $\mathcal {V}$ we define index pairs similarly to the classical case (see Definition 5.1), we prove that $({\text {cl}}S, {\text {mo}}S)$ is one of the possibly many index pairs for *S* (see Proposition 5.3) and we show that the homology of an index pair depends only on *S*, but not on the particular index pair (see Theorem 5.16). This enables us to define the Conley index of an isolated invariant set *S* (see Definition 4.8) and the associated Poincaré polynomial (see (4)). In Example 2.1 (see Fig. 4), the Poincaré polynomials of the isolated invariant sets $S_1=\{A,AC,C,BC,B,AB\}$ and $S_2=\{ABC\}$ are respectively $p_{S_1}(t)=1+t$ and $p_{S_2}(t)=t^2$.

### Morse decompositions

The concept of Morse decomposition in combinatorial dynamics is similar in spirit to the classical case although some details are different (see Definition 7.1). Unlike the classical case, for a combinatorial multivector field $\mathcal {V}$ we prove that the strongly connected components of the directed graph $G_\mathcal {V}$ which admit an essential solution constitute the minimal Morse decomposition of $\mathcal {V}$ (see Theorem 7.3). For Example 2.1 the minimal Morse decomposition consists of six isolated invariant sets:

$$\begin{aligned} M_1 := \{ A, AC, C, BC, B, AB\},\; M_2 := \{ ABC\},\; M_3 := \{ CE\},\\ M_4 := \{ DEF, EF, EFG\},\; M_5 := \{ DF,F,FG\},\; M_6 := \{ G\}. \end{aligned}$$

We say that an isolated invariant set *S* is an *attractor* (respectively a *repeller*) if all solutions originating in it stay in *S* in forward (respectively backward) time (see Sect. 6.1). There are two attractors in our example: $M_1$ is a periodic attractor, and $M_6$ is an attracting stationary point. Sets $M_2$ and $M_4$ are repellers, while $M_3$ and $M_5$ are neither attractors nor repellers.

**Fig. 6:**
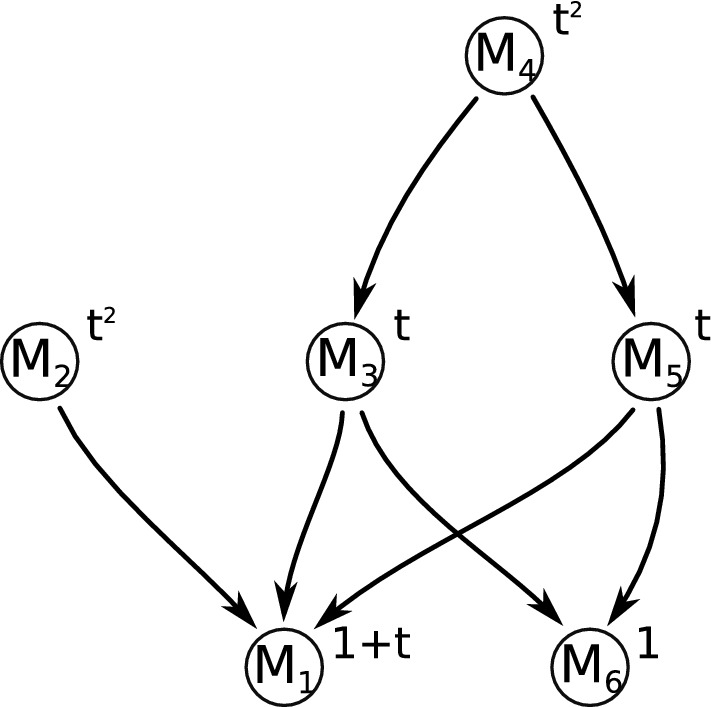
The Conley-Morse graph for the multivector field in Example 2.1

If there exists a path originating in $M_i$ and terminating in $M_j$, we say that there is a *connection* from $M_i$ to $M_j$. The connection relation induces a partial order on Morse sets. The associated poset with nodes labeled with Poincaré polynomials is called the *Conley-Morse graph* of the Morse decomposition, see also Arai et al. (2009); Bush et al. (2012).

The Conley-Morse graph of the minimal Morse decomposition of the combinatorial multivector field in Fig. 4 is presented in Fig. 6. The Morse equation (see Theorem 7.9) for this Morse decomposition takes the form:

$$\begin{aligned} 2t^2 + 3 t + 2 = 1 + (1+t) (1+2t). \end{aligned}$$

As this brief overview of the results of this paper indicates, at least to some extent it is possible to construct a combinatorial analogue of classical topological dynamics. Such an analogue may be used to construct algorithmizable models of sampled dynamical systems as well as tools for computer-assisted proofs in dynamics (Mrozek et al. 2022).

## Preliminaries

In this section we recall the background material needed in this paper and we set notation.

### Sets and maps

We denote the sets of integers, non-negative integers, non-positive integers, and positive integers, respectively, by ${\mathbb {Z}}$, ${\mathbb {Z}}^+$, ${\mathbb {Z}}^-$, and ${\mathbb {N}}$. Given a set *A*, we write $\#A$ for the number of elements in *A* and we denote by $\mathcal {P}(A)$ the family of all subsets of *X*. We write $f:X\nrightarrow Y$ for a partial map from *X* to *Y*, that is, a map defined on a subset ${\text {dom}}{f}\subset X$, called the *domain* of *f*, and such that the set of values of *f*, denoted ${\text {im}}f$, is contained in *Y*.

*A multivalued map* $F: X\multimap Y$ is a map $F: X\rightarrow \mathcal {P}(Y)$ which assigns to every point $x\in X$ a subset $F(x)\subset Y$. Given $A\subset X$, the *image* of *A* under *F* is defined by $F(A):=\bigcup _{x\in A}F(a)$. By the *preimage* of a set $B\subset Y$ with respect to *F* we mean the large preimage, that is,

2 $$\begin{aligned} F^{-1}(B) := \left\{ x\in X\ \mid \ F(x)\cap B\ne \varnothing \right\} . \end{aligned}$$

In particular, if $B=\{y\}$ is a singleton, we get

$$\begin{aligned} F^{-1}(\{y\}) := \left\{ x\in X\ \mid \ y\in F(x)\right\} . \end{aligned}$$

Thus, we have a multivalued map $F^{-1}:Y\multimap X$ given by $F^{-1}(y):=F^{-1}(\{y\})$. We call it the *inverse* of *F*.

### Relations and digraphs

Recall that a binary relation or briefly a relation in a space *X* is a subset $E\subset X\times X$. We write *xEy* as a shorthand for $(x,y)\in E$. The *inverse* of *E* is the relation

$$\begin{aligned} E^{-1}:=\left\{ (y,x)\in X\times X\mid xEy\right\} . \end{aligned}$$

Given a relation *E* in *X*, the pair (*X*, *E*) may be interpreted as a *directed graph* (*digraph*) with vertex set *X*, and edge set *E*.

Relation *E* may also be considered as a multivalued map $E:X\multimap X$ with $E(x):=\{y\in X\mid xEy\}$. Thus, the three concepts: binary relation, multivalued map and directed graph are, in principle, the same and in this paper will be used interchangeably.

We recall that a *path* in a directed graph $G=(X,E)$ is a sequence $x_0, x_1,\dots ,x_k$ of vertices such that $(x_{i-1}, x_i)\in E$ for $i=1,2,\dots k$. The path is *closed* if $x_0 = x_k$. A closed path consisting of two elements is a *loop*. Thus, an $x\in X$ is a loop if and only if $x\in E(x)$. We note that loops may be present at some vertices of *G* but at some other vertices they may be absent.

A vertex is *recurrent* if it belongs to a closed path. In particular, if there is a loop at $x\in X$, then *x* is recurrent. The digraph *G* is *recurrent* if all of its vertices are recurrent. We say that two vertices *x* and *y* in a recurrent digraph *G* are equivalent if there is a path from *x* to *y* and a path from *y* to *x* in *G*. Equivalence of recurrent vertices in a recurrent digraph is easily seen to be an equivalence relation. The equivalence classes of this relation are called *strongly connected components* of digraph *G*. They form a partition of the vertex set of *G*.

We say that a recurrent digraph *G* is *strongly connected* if it has exactly one strongly connected component. A non-empty subset $A\subset X$ is *strongly connected* if $(A, E\cap A\times A)$ is strongly connected. In other words, $A\subset X$ is strongly connected if and only if for all $x,y\in A$ there is a path in *A* from *x* to y and from *y* to *x*.

### Posets

Let *X* be a finite set. We recall that a reflexive and transitive relation $\le$ on *X* is a *preorder* and the pair $(X, \le )$ is a *preordered set*. If $\le$ is also antisymmetric, then it is a *partial order* and $(X,\le )$ is a *poset*. A partial order in which any two elements are comparable is a *linear* (*total*) *order*.

Given a poset $(X,\le )$, a set $A\subset X$ is *convex* if $x\le y\le z$ with $x,z\in A,\ y\in X$ implies $y\in A$. It is an *upper set* if $x\le y$ with $x\in A$ and $y\in X$ implies $y\in A$. Similarly, *A* is a *down set* with respect to $\le$ if $x\le y$ with $y\in A$ and $x\in X$ implies $x\in A$. A *chain* is a totally ordered subset of a poset. Finally, for $A\subset X$ we write

$$\begin{aligned} A^\le&:=\{a\in X\ |\ \exists _{b\in A}\ a\le b\},\\ A^<&:=A^\le \setminus A. \end{aligned}$$

One can easily check the following proposition.

#### Proposition 3.1

([Lipiński 2021, Proposition 1.3.1]) Let $(X,\le )$ be a poset and let $A\subset X$ be a convex set. Then the sets $A^\le$ and $A^<$ are down sets.

### Finite topological spaces

Given a topology $\mathcal {T}$ on *X*, we call $(X,\mathcal {T})$ a topological space. When the topology $\mathcal {T}$ is clear from the context we also refer to *X* as a topological space. We denote the *interior* of $A\subset X$ with respect to $\mathcal {T}$ by ${\text {int}}_\mathcal {T}A$ and the *closure* of *A* with respect to $\mathcal {T}$ by ${\text {cl}}_\mathcal {T}A$. We define the *mouth* of *A* as the set ${\text {mo}}_\mathcal {T}A := {\text {cl}}_\mathcal {T}A\setminus A$. We say that *X* is a *finite topological space* if *X* is a finite set.

If *X* is finite, we also distinguish the *minimal open superset* (or *open hull*) of *A* as the intersection of all the open sets containing *A*. We denote it by ${\text {opn}}_\mathcal {T}A$. We note that when *X* is finite then the family $\mathcal {T}^{{\text {op}}{}}:=\{X\setminus U\mid U\in \mathcal {T}\}$ of closed sets is also a topology on *X*, called *dual* or *opposite topology*. The following proposition is straightforward.

#### Proposition 3.2

If $(X,\mathcal {T})$ is a finite topological space then for every set $A\subset X$ we have ${\text {opn}}_\mathcal {T}A={\text {cl}}_{\mathcal {T}^{{\text {op}}{}}} A$.

If $A=\{a\}$ is a singleton, we simplify the notation ${\text {int}}_\mathcal {T}\{a\}$, ${\text {cl}}_\mathcal {T}\{a\}$, ${\text {mo}}_\mathcal {T}\{a\}$ and ${\text {opn}}_\mathcal {T}\{a\}$ to ${\text {int}}_\mathcal {T}a$, ${\text {cl}}_\mathcal {T}a$, ${\text {mo}}_\mathcal {T}a$ and ${\text {opn}}_\mathcal {T}a$. When the topology $\mathcal {T}$ is clear from the context, we drop the subscript $\mathcal {T}$ in this notation. Given a finite topological space $(X,\mathcal {T})$ we briefly write $X^{{\text {op}}} := (X,\mathcal {T}^{{\text {op}}{}})$ for the same space *X* but with the opposite topology.

We recall that a subset *A* of a topological space *X* is *locally closed* if every $x\in A$ admits a neighborhood *U* in *X* such that $A\cap U$ is closed in *U*. Locally closed sets are important in the sequel. In particular, we have the following characterization of locally closed sets.

#### Proposition 3.3

([Engelking 1989, Problem 2.7.1]) Assume *A* is a subset of a topological space *X*. Then the following conditions are equivalent.

(i) *A* is locally closed,
(ii) ${\text {mo}}_\mathcal {T}A:={\text {cl}}_\mathcal {T}A\setminus A$ is closed in *X*,
(iii) *A* is a difference of two closed subsets of *X*,
(iv) *A* is an intersection of an open set in *X* and a closed set in *X*.

As an immediate consequence of Proposition 3.3(iv) we get the following three propositions.

#### Proposition 3.4

The intersection of a finite family of locally closed sets is locally closed.

#### Proposition 3.5

If *A* is locally closed and *B* is closed, then $A\setminus B$ is locally closed.

#### Proposition 3.6

Let $(X,\mathcal {T})$ be a finite topological space. A subset $A\subset X$ is locally closed in the topology $\mathcal {T}$ if and only if it is locally closed in the topology $\mathcal {T}^{{\text {op}}{}}$.

We recall that the topology $\mathcal {T}$ is $T_2$ or *Hausdorff* if for any two different points $x,y\in X$, there exist disjoint sets $U,V\in \mathcal {T}$ such that $x\in U$ and $y\in V$. It is $T_0$ or *Kolmogorov* if for any two different points $x,y\in X$ there exists a $U\in \mathcal {T}$ such that $U\cap \{x,y\}$ is a singleton.

Finite topological spaces stand out from general topological spaces by the fact that the only Hausdorff topology on a finite topological space *X* is the discrete topology consisting of all subsets of *X*.

#### Proposition 3.7

([Lipiński 2021, Proposition 1.4.7]) Let $(X,\mathcal {T})$ be a finite topological space and $A\subset X$. Then ${\text {cl}}A =\bigcup _{a\in A}{\text {cl}}a.$

A remarkable feature of finite topological spaces is the following theorem.

#### Theorem 3.8

(Alexandrov 1937) For a preorder $\le$ on a finite set *X*, there is a topology $\mathcal {T}_\le$ on *X* whose open sets are the upper sets with respect to $\le$. For a topology $\mathcal {T}$ on a finite set *X*, there is a preorder $\le _\mathcal {T}$ where $x\le _\mathcal {T}y$ if and only if $x\in {\text {cl}}_\mathcal {T}y$. The correspondences $\mathcal {T}\mapsto \;\le _\mathcal {T}$ and $\le \;\mapsto \mathcal {T}_\le$ are mutually inverse. Under these correspondences continuous maps are transformed into order-preserving maps and vice versa. Moreover, the topology $\mathcal {T}$ is $T_0$ (Kolmogorov) if and only if the preorder $\le _\mathcal {T}$ is a partial order.

The correspondence resulting from Theorem 3.8 provides a method to translate concepts and problems between topology and order theory in finite spaces. In particular, closed sets are translated to down sets in this correspondence and we have the following straightforward proposition.

#### Proposition 3.9

Let $(X,\mathcal {T})$ be a finite topological space. Then, for $A\subset X$ we have

$$\begin{aligned} {\text {cl}}_\mathcal {T}A&= \{x\in X\mid \exists _{a\in A}\ x\le _\mathcal {T}a\},\\ {\text {opn}}_\mathcal {T}A&= \{x\in X\mid \exists _{a\in A}\ x\ge _\mathcal {T}a\},\\ {\text {int}}_\mathcal {T}A&= \{a\in A\mid \forall _{x\in X}\ x\ge _\mathcal {T}a\ \Rightarrow x\in A\}. \end{aligned}$$

In other words, ${\text {cl}}_\mathcal {T}A$ is the minimal down set with respect to $\le _\mathcal {T}$ containing *A*, ${\text {opn}}_\mathcal {T}A$ is the minimal upper set with respect to $\le _\mathcal {T}$ containing *A* and ${\text {int}}_\mathcal {T}A$ is the maximal upper set with respect to $\le _\mathcal {T}$ contained in *A*.

One can easily verify the following proposition.

#### Proposition 3.10

([Lipiński 2021, Proposition 1.4.10]) Assume *X* is a $T_0$ finite topological space and $A\subset X$. Then *A* is locally closed if and only if *A* is convex with respect to $\le _\mathcal {T}$.

### Lefschetz complexes

We say that $(X,\kappa )$ is a *Lefschetz complex* (see [Lefschetz 1942, Chapter III, Sect. 1, Definition 1.1]) if $X=(X_q)_{q\in {\mathbb {Z}^+}}$ is a finite set with gradation, $\kappa : X \times X \rightarrow R$ is a map with values in a ring with unity such that $\kappa (x,y)\ne 0$ implies both the inclusion $x\in X_q$ and $y\in X_{q-1}$, and for any $x,z\in X$ we have

3 $$\begin{aligned} \sum _{y\in X}\kappa (x,y)\kappa (y,z)=0. \end{aligned}$$

One easily verifies that by condition (3) we have a free chain complex $(R(X),\partial ^\kappa )$ with $\partial ^\kappa :R(X)\rightarrow R(X)$ defined on generators by $\partial ^\kappa (x) := \sum _{y\in X}\kappa (x,y)y$. The *Lefschetz homology* of $(X,\kappa )$, denoted $H^\kappa (X)$, is the homology of this chain complex.

Note that the elements of *X* may be identified with a basis of *R*(*X*). Vice versa, every fixed basis of a finitely generated chain complex constitutes a Lefschetz complex.

Given $x,y\in X$ we say that *y* is a *facet* of *x* if $\kappa (x,y)\ne 0$. It is easily seen that the facet relation extends uniquely to a minimal partial order. Via the Alexandrov Theorem (Alexandrov 1937), this partial order makes every Lefschetz complex a finite topological space.

### Homology in finite topological spaces

In the sequel we need a homology theory in finite topological spaces. Singular homology (Munkres 1984) is well defined for any topological space, in particular for a finite topological space. McCord’s Theorem (McCord 1966) states that every finite topological space is weakly homotopy equivalent to the associated order complex $\mathcal {K}(X)$, that is, an abstract simplicial complex consisting of subsets of *X* linearly ordered by the partial order associated with the topology via the Alexandrov Theorem (Theorem 3.8). This is convenient for computational purposes.

For Lefschetz complexes, apart from singular homology we also have the notion of Lefschetz homology. As we already mentioned in Sect. 2.1 the singular homology and Lefschetz homology of a Lefschetz complex need not be the same. Nevertheless, they both satisfy the following theorem which summarizes the features of homology we need in this paper.

#### Theorem 3.11

Let $(X,\mathcal {T})$ be a finite topological space, and for a closed subset $A\subset X$ let *H*(*X*, *A*) denote the singular homology of the pair (*X*, *A*). Then the following properties hold.

(i) If *A*, *B*, *C*, *D* are closed subsets of *X* such that $B\subset A$, $D\subset C$ and $A \setminus B = C \setminus D$, then $H(A, B) \cong H(C, D)$.
(ii) If $B\subset A\subset X$ are closed, then the inclusions induce the exact sequence
  $$\begin{aligned} \dotsc \rightarrow H_n(A,B)\rightarrow H_n(X,B)\rightarrow H_n(X,A)\rightarrow H_{n-1}(A,B)\rightarrow \dotsc . \end{aligned}$$
(iii) If $Y_0\subset X_0\subset X$ and $Y_1\subset X_1\subset X$ are closed in *X* then there is an inclusion induced exact sequence
  $$\begin{aligned} \dotsc \rightarrow H_n(X_0\cap X_1, Y_0\cap Y_1)\rightarrow H_n(X_0,Y_0)\oplus H_n(X_1,Y_1)\rightarrow \\ H_n(X_0\cup X_1, Y_0\cup Y_1)\rightarrow H_{n-1}(X_0\cap X_1, Y_0\cap Y_1)\dotsc . \end{aligned}$$

Moreover, if *X* is a Lefschetz complex, then properties (i), (ii), (iii) above also hold with singular homology replaced by Lefschetz homology.

#### Proof

In the case of a Lefschetz complex and Lefschetz homology all three properties are easy exercises in homological algebra. Property (ii) for singular homology is standard [Munkres 1984, Theorem 30.2]. Property (i) follows from the excision property of simplicial homology [Munkres 1984, Theorem 9.1] via McCord’s Theorem (McCord 1966) (compare Lipiński 2021). Similarly, property (iii) for singular homology can be established in the same way from the relative simplicial Mayer-Vietoris sequence [Munkres 1984, Chapter 25 Ex.2]. $\square$

In the sequel we fix a homology theory which satisfies properties (i)-(iii) of Theorem 3.11. This may be singular homology in the case of an arbitrary finite topological space or the Lefschetz homology in the case of a Lefschetz complex. Clearly, both Lefschetz homology and singular homology for finite spaces are finitely generated. In consequence, for a locally closed $A\subset X$ the *Poincaré formal power series*

4 $$\begin{aligned} p_A(t):=\sum _{i=0}^\infty \beta _i(A)t^i \end{aligned}$$

with $\beta _i(A):={\text {rank}}H_i({\text {cl}}A,{\text {mo}}A)$ denoting *i*th *Betti number*, is well defined and a polynomial.

## Dynamics of combinatorial multivector fields

In this section we introduce and study the main concepts of combinatorial topological dynamics studied in this paper: isolated invariant sets of combinatorial multivector fields.

### Multivalued dynamical systems in finite spaces

By a *combinatorial dynamical system* or briefly, a *dynamical system* in a finite space *X* we mean a multivalued map $\Pi :X\times {\mathbb {Z}}^+ \multimap X$ such that

5 $$\begin{aligned} \Pi \left( \Pi (x,m),n\right) =\Pi (x,m+n) \quad \text{ for } \text{ all }\quad m,n \in {\mathbb {Z}}^+, \; x \in X. \end{aligned}$$

Let $\Pi$ be a combinatorial dynamical system. Consider the multivalued map $\Pi ^n:X\multimap X$ given by $\Pi ^n(x):=\Pi (x,n)$. We call $\Pi ^1$ the *generator* of the dynamical system $\Pi$. It follows from (5) that the combinatorial dynamical system $\Pi$ is uniquely determined by its generator. Thus, it is natural to identify a combinatorial dynamical system with its generator. In particular, we consider any multivalued map $\Pi : X\multimap X$ as a combinatorial dynamical system $\Pi : X\times {\mathbb {Z}}^+ \multimap X$ defined recursively by

$$\begin{aligned} \Pi (x,1)&:=\Pi (x),\\ \Pi (x,n+1)&:=\Pi (\Pi (x, n)), \end{aligned}$$

as well as $\Pi (x,0) := x$. We call it the combinatorial dynamical system induced by a map $\Pi$. In particular, the inverse $\Pi ^{-1}$ of $\Pi$ also induces a combinatorial dynamical system. We call it the *dual dynamical system*.

### Solutions and paths

By a ${\mathbb {Z}}$-interval we mean a set of the form ${\mathbb {Z}}\cap I$ where *I* is an interval in ${\mathbb {R}}$. A ${\mathbb {Z}}$-interval is *left bounded* if it has a minimum; otherwise it is *left-infinite*. It is *right bounded* if it has a maximum; otherwise it is *right-infinite*. It is *bounded* if it has both a minimum and a maximum. It is *unbounded* if it is not bounded.

A *solution* of a combinatorial dynamical system $\Pi :X\multimap X$ in $A\subset X$ is a partial map $\varphi :{\mathbb {Z}}\nrightarrow A$ whose *domain*, denoted ${\text {dom}}\varphi$, is a ${\mathbb {Z}}$-interval and for any $i,i+1\in {\text {dom}}\varphi$ the inclusion $\varphi (i+1)\in \Pi (\varphi (i))$ holds. The solution *passes* through $x\in X$ if $x=\varphi (i)$ for some $i\in {\text {dom}}\varphi$. The solution $\varphi$ is *full* if ${\text {dom}}\varphi ={\mathbb {Z}}$. It is a *backward solution* if ${\text {dom}}\varphi$ is left-infinite. It is a *forward solution* if ${\text {dom}}\varphi$ is right-infinite. It is a *partial solution* or simply a *path* if ${\text {dom}}\varphi$ is bounded.

A full solution $\varphi :{\mathbb {Z}}\rightarrow X$ is *periodic* if there exists a $T\in {\mathbb {N}}$ such that $\varphi (t+T) =\varphi (t)$ for all $t\in {\mathbb {Z}}$. Note that every closed path may be extended to a periodic solution.

If the maximum of ${\text {dom}}\varphi$ exists, we call the value of $\varphi$ at this maximum the *right endpoint* of $\varphi$. If the minimum of ${\text {dom}}\varphi$ exists, we call the value of $\varphi$ at this minimum the *left endpoint* of $\varphi$. We denote the left and right endpoints of $\varphi$, respectively, by $\varphi ^{\sqsubset }$ and $\varphi ^{\sqsupset }$.

By a *shift* of a solution $\varphi$ we mean the composition $\varphi \circ \tau _n$, where the map $\tau _n:{\mathbb {Z}}\ni m\mapsto m+n\in {\mathbb {Z}}$ is translation. Given two solutions $\varphi$ and $\psi$ such that $\psi ^{\sqsubset }$ and $\varphi ^{\sqsupset }$ exist and $\psi ^{\sqsubset }\in \Pi (\varphi ^{\sqsupset })$, there is a unique shift $\tau _n$ such that $\varphi \cup (\psi \circ \tau _n$) is a solution. We call this union of paths the *concatenation* of $\varphi$ and $\psi$ and we denote it by $\varphi \cdot \psi$. We also identify each $x\in X$ with the trivial solution $\varphi :\{0\}\rightarrow \{x\}$. For a full solution $\varphi$ we denote the restrictions $\left. \varphi \right| _{{\mathbb {Z}}^+}$ by $\varphi ^+$ and $\left. \varphi \right| _{{\mathbb {Z}}^-}$ by $\varphi ^-$. We finish this section with the following straightforward proposition.

#### Proposition 4.1

If $\varphi :{\mathbb {Z}}\rightarrow X$ is a full solution of a dynamical system $\Pi :X\multimap X$, then ${\mathbb {Z}}\ni t\rightarrow \varphi (-t)\in X$ is a solution of the dual dynamical system induced by $\Pi ^{-1}$. We call it the *dual solution* and denote it $\varphi ^{{\text {op}}}$.

### Combinatorial multivector fields

Combinatorial multivector fields on Lefschetz complexes were introduced in [Mrozek 2017, Definition 5.10]. In this paper, we generalize this definition as follows. Let $(X,\mathcal {T})$ be a finite topological space. By a *combinatorial multivector* in *X* we mean a locally closed and non-empty subset of *X*. We define a *combinatorial multivector field* as a partition $\mathcal {V}$ of *X* into multivectors. Therefore, unlike (Mrozek 2017), we do not assume that a multivector has a unique maximal element with respect to $\le _\mathcal {T}$. Such an assumption was introduced in Mrozek (2017) for technical reasons but it is very inconvenient in applications. As an example we mention the following straightforward proposition which is not true in the setting of Mrozek (2017).

#### Proposition 4.2

Assume $\mathcal {V}$ is a combinatorial multivector field on a finite topological space *X* and $Y\subset X$ is a locally closed subspace. Then

$$\begin{aligned} \mathcal {V}_Y:={\{\,V\cap Y\mid V\in \mathcal {V}, V\cap Y\ne \varnothing \,\}} \end{aligned}$$

is a multivector field in *Y*. We call it the *multivector field induced by* $\mathcal {V}$. $\square$

We say that a multivector *V* is *critical* if the relative homology $H({\text {cl}}V,{\text {mo}}V)$ is non-zero. A multivector *V* which is not critical is called *regular*. For each $x\in X$ we denote by $[x]_{\mathcal {V}}$ the unique multivector in $\mathcal {V}$ which contains *x*. If the multivector field $\mathcal {V}$ is clear from the context, we write briefly $[x]:=[x]_{\mathcal {V}}$.

We say that $x\in X$ is *critical* (respectively *regular*) with respect to $\mathcal {V}$ if $[x]_{\mathcal {V}}$ is critical (respectively regular). We say that a subset $A\subset X$ is $\mathcal {V}$-*compatible* if for each $x\in X$ either $[x]_{\mathcal {V}}\cap A=\varnothing$ or $[x]_{\mathcal {V}}\subset A$. Note that every $\mathcal {V}$-compatible set $A\subset X$ induces a well-defined multivector field $\mathcal {V}_A:=\{V\in \mathcal {V}\mid V\subset A\}$ on *A*. The next proposition follows immediately from the definition of a $\mathcal {V}$-compatible set.

#### Proposition 4.3

The union and the intersection of a family of $\mathcal {V}$-compatible sets is $\mathcal {V}$-compatible.

We associate with every multivector field $\mathcal {V}$ a combinatorial dynamical system on *X* induced by the multivalued map $\Pi _\mathcal {V}: X\multimap X$ given by

6 $$\begin{aligned} \Pi _\mathcal {V}(x):= [x]_{\mathcal {V}}\cup {\text {cl}}x. \end{aligned}$$

The following proposition is straightforward.

#### Proposition 4.4

Let $\mathcal {V}$ be a multivector field on *X*. Then

$$\begin{aligned} \Pi _\mathcal {V}(x) = [x]_{\mathcal {V}}\cup {\text {mo}}x. \end{aligned}$$

**Fig. 7:**
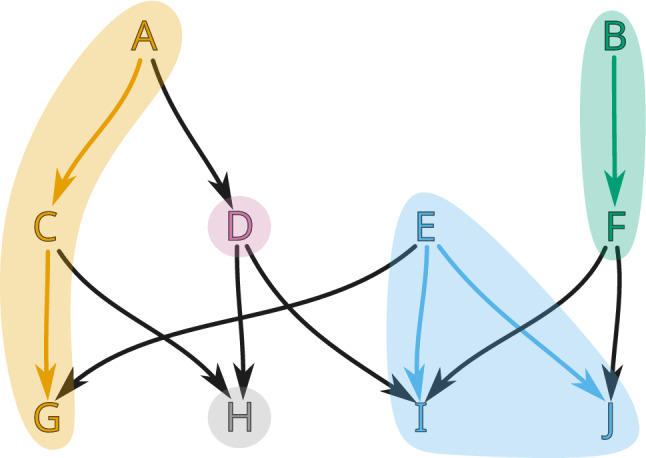
An example of a combinatorial multivector field $\mathcal {V}=\{\{A,C,G\}, \{D\}, \{H\}, \{E,I,J\}, \{B,F\}\}$ on a finite topological space consisting of ten points. There are two regular multivectors, $\{A,C,G\}$ and $\{E,I,J\}$, the others are critical. Both the nodes and the connecting edges of each multivector are highlighted with a different color

One can also easily prove the following proposition.

#### Proposition 4.5

([Lipiński 2021, Proposition 4.1.5]) Let $\mathcal {V}$ be a combinatorial multivector field on $(X,\mathcal {T})$. If $A\subset X$, then

$$\begin{aligned} \Pi _\mathcal {V}^{-1}(A)=\bigcup _{x\in A} [x]_\mathcal {V}\cup {\text {opn}}x. \end{aligned}$$

**Fig. 8:**
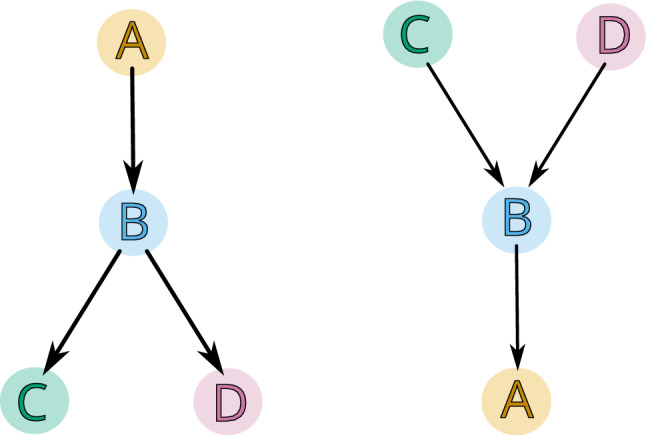
An example of a finite topological space *X* and $X^{{\text {op}}{}}$ consisting of four points and with the same partition into multivectors $\mathcal {V}=\{\{A\}, \{B\}, \{C\}, \{D\}\}$. In *X* multivectors $\{B\}$, $\{C\}$ and $\{D\}$ are critical, while in $X^{{\text {op}}{}}$ only $\{A\}$ is critical

Note that by Proposition 3.6 a multivector in a finite topological space *X* is also a multivector in $X^{{\text {op}}{}}$, that is, in the space *X* with the opposite topology. Thus, a multivector field $\mathcal {V}$ in *X* is also a multivector field in $X^{{\text {op}}{}}$. However, the two multivector fields cannot be considered the same, because the change in topology implies the change of the location of critical and regular multivectors (see Fig. 8). We indicate this in notation by writing $\mathcal {V}^{{\text {op}}{}}$ for the multivector field $\mathcal {V}$ considered with the opposite topology.

The multivector field $\mathcal {V}^{{\text {op}}{}}$ induces a combinatorial dynamical system $\Pi _{\mathcal {V}^{{\text {op}}{}}}:X^{{\text {op}}{}}\multimap X^{{\text {op}}{}}$ given by $\Pi _{\mathcal {V}^{{\text {op}}{}}}(x):=[x]_\mathcal {V}\cup {\text {cl}}_{\mathcal {T}^{{\text {op}}{}}} x$. As an immediate consequence of Proposition 3.2 and Proposition 4.5 we get following result.

#### Proposition 4.6

The combinatorial dynamical system $\Pi _\mathcal {V}^{{\text {op}}{}}$ is dual to the combinatorial dynamical system $\Pi _\mathcal {V}$, that is, we have $\Pi _\mathcal {V}^{{\text {op}}{}}=\Pi _\mathcal {V}^{-1}$.

### Essential solutions

Given a multivector field $\mathcal {V}$ on a finite topological space *X* by a solution (full solution, forward solution, backward solution, partial solution or path) of $\mathcal {V}$ we mean a corresponding solution type of the combinatorial dynamical system $\Pi _\mathcal {V}$. Given a solution $\varphi$ of $\mathcal {V}$ we denote by $\mathcal {V}(\varphi )$ the set of multivectors $V\in \mathcal {V}$ such that $V\cap {\text {im}}\varphi \ne \varnothing$. We denote the set of all paths of $\mathcal {V}$ in a set *A* by ${\text {Path}}_\mathcal {V}(A)$ and define

$$\begin{aligned} {\text {Path}}_\mathcal {V}(x, A)&:= \{\varphi \in {\text {Path}}_\mathcal {V}(A)\ \mid \ \varphi (0)=x\},\\ {\text {Path}}_\mathcal {V}(x, y, A)&:= \{\varphi \in {\text {Path}}_\mathcal {V}(A)\ \mid \ \varphi ^{\sqsubset }=x\ \text {and}\ \varphi ^{\sqsupset }=y\}. \end{aligned}$$

We denote the set of full solutions of $\mathcal {V}$ in *A* (respectively backward or forward solutions in *A*) by ${\text {Sol}}_\mathcal {V}(A)$ (respectively ${\text {Sol}}_\mathcal {V}^-(A)$, ${\text {Sol}}_\mathcal {V}^+(A)$). We also write ${\text {Sol}}_\mathcal {V}(x,A) := \{\varphi \in {\text {Sol}}_\mathcal {V}(A) \mid \varphi (0)=x\}.$ Observe that by (6) $x\in \Pi _\mathcal {V}(x)$ for every $x\in X$. Hence, a constant map from an interval to a point is always a solution. This means that every solution can easily be extended to a full solution. In consequence, every point is recurrent which is not typical. To remedy this we introduce the concept of an essential solution.

A full solution $\varphi :{\mathbb {Z}}\rightarrow X$ is *left-essential* (respectively *right-essential*) if for every regular $x\in {\text {im}}\varphi$ the set ${\{\,t\in {\mathbb {Z}}\mid \varphi (t)\not \in [x]_{\mathcal {V}}\,\}}$ is *left-infinite* (respectively *right-infinite*). We say that $\varphi$ is *essential* if it is both left- and right-essential. We say that a point $x\in X$ is *essentially recurrent* if an essential periodic solution passes through *x*. Note that a periodic solution $\varphi$ is essential either if $\#\mathcal {V}(\varphi )\ge 2$ or if the unique multivector in $\mathcal {V}(\varphi )$ is critical.

We denote the set of all essential solutions in $A\subset X$ (respectively left- or right-essential solutions in *A*) by ${\text {eSol}}_\mathcal {V}(A)$ (respectively ${\text {eSol}}_\mathcal {V}^+(A)$, ${\text {eSol}}_\mathcal {V}^-(A)$) and the set of all essential solutions in a set $A\subset X$ passing through a point *x* by ${\text {eSol}}_\mathcal {V}(x,A) := \{\varphi \in {\text {eSol}}(A) \mid \varphi (0)=x\}$ and we define the *invariant part* of $A\subset X$ by

7 $$\begin{aligned} {\text {Inv}}_\mathcal {V}A := \left\{ x\in A\ |\ {\text {eSol}}(x,A)\ne \varnothing \right\} . \end{aligned}$$

In particular, if ${\text {Inv}}_\mathcal {V}A = A$ then we say that *A* is an *invariant set* for $\mathcal {V}$. We drop the subscript $\mathcal {V}$ in ${\text {Sol}}_\mathcal {V}$, ${\text {eSol}}_\mathcal {V}$ and ${\text {Inv}}_\mathcal {V}$ whenever $\mathcal {V}$ is clear from the context.

#### Proposition 4.7

Let $A,B\subset X$ be invariant sets. Then $A\cup B$ is also an invariant set.

#### Proof

Let $x\in A$. By the definition of an invariant set there exists an essential solution $\varphi \in {\text {eSol}}(x,A)$. It is clear that $\varphi$ is also an essential solution in $A\cup B$. Thus ${\text {eSol}}(x,A\cup B)\ne \varnothing$. The same holds for *B*. Hence ${\text {Inv}}(A\cup B)=A\cup B$. $\square$

### Isolated invariant sets

In this subsection we introduce the combinatorial counterpart of the concept of an isolated invariant set. In order to emphasize the difference, we say that an isolated invariant set is isolated by an isolating set, not by an isolating neighborhood. In comparison to the classical theory of dynamical systems, the crucial difference is that we cannot guarantee the existence of disjoint isolating sets for two disjoint isolated invariant sets. This is caused by the tightness of the finite topological space.

#### Definition 4.8

A closed set *N* *isolates* an invariant set $S\subset N$, if the following two conditions hold:

- Every path in *N* with endpoints in *S* is a path in *S*,
- $$\Pi _\mathcal {V}(S)\subset N$$.

In this case, we also say that *N* is an *isolating set* for *S*. An invariant set *S* is *isolated* if there exists a closed set *N* meeting the above conditions.

An important example is given by the following straightforward proposition.

#### Proposition 4.9

The whole space *X* isolates its invariant part ${\text {Inv}}X$. In particular, ${\text {Inv}}X$ is an isolated invariant set. $\square$

#### Proposition 4.10

If $S\subset X$ is an isolated invariant set, then *S* is $\mathcal {V}$-compatible.

#### Proof

Suppose the contrary. Then there exists an $x\in S$ and a $y\in [x]_\mathcal {V}\setminus S$. Let *N* be an isolating set for *S*. It follows from Definition 4.8(b), that $y\in \Pi _\mathcal {V}(x)\subset N$. It is also clear that $x\in \Pi _\mathcal {V}(y)$. Thus the path $x\cdot y\cdot x$ is a path in *N* with endpoints in *S*, but it is not contained in *S*, and this in turn contradicts Definition 4.8(a). $\square$

The finiteness of the space allows us to construct the smallest possible isolating set. More precisely, we have the following straightforward proposition.

#### Proposition 4.11

Let *N* be an isolating set for an isolated invariant set *S*. If *M* is a closed set such that $S\subset M\subset N$, then *S* is also isolated by *M*. In particular, ${\text {cl}}S$ is the smallest isolating set for *S*. $\square$

#### Proposition 4.12

Let $S \subset X$. If *S* is an isolated invariant set, then *S* is locally closed.

#### Proof

By Proposition 4.11 the set $N := {\text {cl}}S$ is an isolating set for *S*. Assume that *S* is not locally closed. By Proposition 3.10 there exist $x, z \in S$ and a $y \not \in S$ such that $x \le _\mathcal {T}y \le _\mathcal {T}z$. Hence, it follows from Theorem 3.8 that $x\in {\text {cl}}_\mathcal {T}y$ and $y\in {\text {cl}}_\mathcal {T}z$. In particular, $x,y,z\in {\text {cl}}S$. It follows that $\varphi :=z\cdot y\cdot x$ is a solution in ${\text {cl}}S$ with endpoints in *S*. In consequence, $y\in S$, a contradiction. $\square$

In particular, it follows from Proposition 4.12 that if *S* is an isolated invariant set, then we have the induced multivector field $\mathcal {V}_S$ on *X*.

#### Proposition 4.13

Let *S* be a locally closed, $\mathcal {V}$-compatible invariant set. Then *S* is an isolated invariant set.

#### Proof

Assume that *S* is a $\mathcal {V}$-compatible and locally closed invariant set. We will show that $N:={\text {cl}}S$ isolates *S*. We have

$$\begin{aligned} \Pi _\mathcal {V}(S) = \bigcup _{x\in S} {\text {cl}}x \cup \bigcup _{x\in S} [x]_\mathcal {V}= {\text {cl}}S \cup S = {\text {cl}}S \subset N. \end{aligned}$$

Therefore condition (b) of Definition 4.8 is satisfied.

We will now show that every path in *N* with endpoints in *S* is a path in *S*. Let $\varphi :=x_0\cdot x_1\cdot ...\cdot x_n$ be a path in *N* with endpoints in *S*. Thus, $x_0, x_n\in S$. Suppose that there is an $i\in \{0,1,...,n\}$ such that $x_i\not \in S$. Without loss of generality we may assume that *i* is maximal such that $x_i\not \in S$. Then $x_{i+1}\ne x_i$ and $i<n$, because $x_n\in S$. We have $x_{i+1}\in \Pi _\mathcal {V}(x_i)=[x_i]_\mathcal {V}\cup {\text {cl}}x_i$. Since $x_i\not \in S$, $x_{i+1}\in S$ and *S* is $\mathcal {V}$-compatible, we cannot have $x_{i+1}\in [x_i]_\mathcal {V}$. Therefore, $x_{i+1}\in {\text {cl}}x_i$. Since $\varphi$ is a path in $N={\text {cl}}{S}$, we have $x_i\in {\text {cl}}S$. Hence, $x_i\in {\text {cl}}z$ for a $z\in S$. It follows from Proposition 3.10 that $x_i\in S$, because $x_{i+1},z\in S$, $x_{i+1}\in {\text {cl}}x_i$, $x_i\in {\text {cl}}z$ and *S* is locally closed. Thus, we get a contradiction proving that also condition (a) of Definition 4.8 is satisfied. In consequence, *N* isolates *S* and *S* is an isolated invariant set. $\square$

### Multivector field as a digraph

Let $\mathcal {V}$ be a multivector field in *X*. We denote by $G_\mathcal {V}$ the multivalued map $\Pi _\mathcal {V}$ interpreted as a digraph.

#### Proposition 4.14

Assume $A\subset X$ is strongly connected in $G_\mathcal {V}$. Then the following conditions are pairwise equivalent.

(i) There exists an essentially recurrent point *x* in *A*, that is, there exists an essential periodic solution in *A* through *x*,
(ii) *A* is non-empty and every point in *A* is essentially recurrent in *A*,
(iii) $${\text {Inv}}A\ne \varnothing $$.

#### Proof

Assume (i). Then $A\ne \varnothing$. Let $x\in A$ be an essentially recurrent point in *A* and let $y\in A$ be arbitrary. Since *A* is strongly connected, we can find a periodic solution $\varphi$ in *A* passing through *x* and *y*. If $\#\mathcal {V}(\varphi )\ge 2$, then $\varphi$ is essential and *y* is essentially recurrent in *A*. Otherwise $[x]_\mathcal {V}=[y]_\mathcal {V}$ and we may easily modify the essential periodic solution in *A* through *x* to an essential periodic solution in *A* through *y*. This proves (ii). Implication (ii)$\Rightarrow$(iii) is straightforward. To prove that (iii) implies (i) assume that $\varphi$ is an essential solution in *A*, If $\#\mathcal {V}(\varphi )=1$, then the unique multivector $V\in \mathcal {V}(\varphi )$ is critical and every $x\in V\subset A$ is essentially recurrent in *A*. Otherwise we can find points $x,y\in A$ such that $[x]_\mathcal {V}\ne [y]_\mathcal {V}$. Since *A* is strongly connected, we can find paths $\psi _1\in {\text {Path}}_\mathcal {V}(x,y,A)$ and $\psi _2\in {\text {Path}}_\mathcal {V}(y,x,A)$. Then $\psi _1\cdot \psi _2$ extends to an essential periodic solution in *A* through *x* proving that $x\in A$ is essentially recurrent in *A*. $\square$

The above result considered the situation of a strongly connected set in $G_\mathcal {V}$. If in addition we assume that this set is maximal, that is, a strongly connected component in $G_\mathcal {V}$, we obtain the following result.

#### Proposition 4.15

Let $\mathcal {V}$ be a multivector field on *X* and let $G_\mathcal {V}$ be the associated digraph. If $C\subset X$ is a strongly connected component of $G_\mathcal {V}$, then *C* is $\mathcal {V}$-compatible and locally closed.

#### Proof

Let $x\in C$ and $y\in [x]_{\mathcal {V}}$. It is clear that $x\cdot y\in {\text {Path}}_\mathcal {V}(x,y,X)$ and $y\cdot x\in {\text {Path}}_\mathcal {V}(y,x,X)$. Hence *C* is $\mathcal {V}$-compatible.

Let $x,z\in C$, $y\in X$ be such that $x\le _\mathcal {T}y\le _\mathcal {T}z$. Since *C* is strongly connected we can find a path $\rho$ from *x* to *z*. Clearly, by Proposition 3.9 and (6) we have $y\in \Pi _\mathcal {V}(z)$ and $x\in \Pi _\mathcal {V}(y)$. Thus $y\cdot \rho \in {\text {Path}}_\mathcal {V}(y,z,X)$ and $z\cdot y\in {\text {Path}}_\mathcal {V}(z,y,X)$. It follows that $y\in C$. Hence, *C* is convex and, by Proposition 3.10, C is locally closed. $\square$

The following theorem shows that some isolated invariant sets of combinatorial multivector fields may be characterized in purely graph-theoretic terms. However, it is not difficult to give examples of isolated invariant sets which cannot be characterized this way (see [25]).

#### Theorem 4.16

Let $\mathcal {V}$ be a multivector field on *X* and let $G_\mathcal {V}$ be the associated digraph. If $C\subset X$ is a strongly connected component of $G_\mathcal {V}$ such that ${\text {eSol}}(C)\ne \varnothing$, then *C* is an isolated invariant set.

#### Proof

According to Proposition 4.13 it suffices to prove that *C* is a $\mathcal {V}$-compatible, locally closed invariant set. It follows from Proposition 4.15 that *C* is $\mathcal {V}$-compatible and locally closed. Thus, we only need to show that *C* is invariant. Since ${\text {Inv}}C\subset C$, we only need to prove that $C\subset {\text {Inv}}C$. Let $y\in C$. Since ${\text {eSol}}(C)\ne \varnothing$, we may take an $x\in C$ and a $\varphi \in {\text {eSol}}(x,C)$. Since *C* is strongly connected we can find paths $\rho$ and $\rho '$ in *C* from *x* to *y* and from *y* to *x* respectively. Then the solution $\varphi ^-\cdot \rho \cdot \rho '\cdot \varphi ^+$ is a well-defined essential solution through *y* in *C*. Thus, ${\text {eSol}}(y,C)\ne \varnothing$, which proves that we have $y\in {\text {Inv}}C$. $\square$

**Fig. 9:**
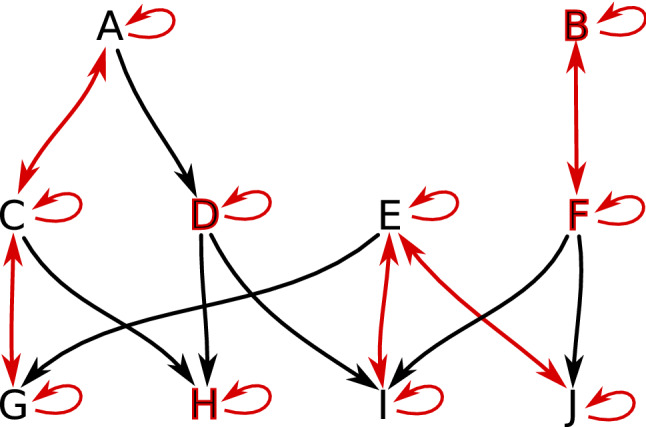
Digraph $G_\mathcal {V}$ for a multivector field from Fig. 7. Black edges are induced by closure relation, while the red bi-directional edges represent connections within a multivector. For clarity, we omit the edges that can be obtained by the between-level transitivity (e.g., from *A* to *G*). Nodes that are part of a critical multivector are additionally bolded in red

## Index pairs and Conley index

In this section we construct the Conley index of an isolated invariant set of a combinatorial multivector field. As in the classical case we define index pairs, prove their existence and prove that the homology of an index pair depends only on the isolated invariant set and not on the choice of index pair.

### Index pairs and their properties

#### Definition 5.1

Let *S* be an isolated invariant set. A pair $P=(P_1, P_2)$ of closed subsets of *X* such that $P_2\subset P_1$, is called an *index pair for* *S* if

1. $$x \in P_2,\ y \in \Pi _\mathcal {V}(x) \cap P_1\ \Rightarrow \ y \in P_2$$ (positive invariance),
2. $$x \in P_1,\ \Pi _\mathcal {V}(x) \setminus P_1 \ne \varnothing \ \Rightarrow \ x \in P_2$$ (exit set),
3. $$S = {\text {Inv}}(P_1\setminus P_2)$$ (invariant part).

An index pair *P* is said to be *saturated* if $S=P_1\setminus P_2$.

#### Proposition 5.2

Let *P* be an index pair for an isolated invariant set *S*. Then $P_1$ isolates *S*.

#### Proof

According to our assumptions, the set $P_1$ is closed, and we clearly have $S={\text {Inv}}(P_1\setminus P_2)\subset P_1\setminus P_2\subset P_1$. Thus, it only remains to be shown that conditions (a) and (b) in Definition 4.8 are satisfied.

Suppose there exists a path $\psi :=x_0\cdot x_1\cdot \dotsc \cdot x_n$ in $P_1$ such that $x_0, x_n\in S$ and $x_i\in P_1\setminus S$ for some $i\in \{1,2,\dotsc ,n-1\}$. First, we will show that ${\text {im}}\psi \subset P_1\setminus P_2$. To this end, suppose the contrary. Then, there exists an $i\in \{1,2,\dotsc ,n-1\}$ such that $x_i\in P_2$ and $x_{i+1}\in P_1\setminus P_2$. Since $\psi$ is a path we have $x_{i+1}\in \Pi _\mathcal {V}(x_i)$. But, (IP1) implies $x_{i+1}\in P_2$, a contradiction.

Since *S* is invariant and $x_0,x_n\in S$, we may take a $\varphi _0\in {\text {eSol}}(x_0, S)$ and a $\varphi _n\in {\text {eSol}}(x_n, S)$. The solution $\varphi _0^-\cdot \psi \cdot \varphi _n^+$ is an essential solution in $P_1\setminus P_2$ through $x_i$. Thus, $x_i\in {\text {Inv}}(P_1\setminus P_2)=S$, a contradiction. This proves that every path in $P_1$ with endpoints in *S* is contained in *S*, and therefore Definition 4.8(a) is satisfied.

In order to verify (b), let $x \in S$ be arbitrary. We have already seen that then $x \in P_1 \setminus P_2 \subset P_1$. Now suppose that $\Pi _\mathcal {V}(x) \setminus P_1 \ne \varnothing$. Then (IP2) implies $x \in P_2$, which contradicts $x \in P_1 \setminus P_2$. Therefore, we necessarily have $\Pi _\mathcal {V}(x) \setminus P_1 = \varnothing$, that is, $\Pi _\mathcal {V}(x) \subset P_1$, which immediately implies (b). Hence, $P_1$ isolates *S*. $\square$

One can easily see from the above proof that in Definition 5.1 one does not have to assume that *S* is an isolated invariant set. In fact, the proof of Proposition 5.2 implies that any invariant set which admits an index pair is automatically an isolated invariant set. Furthermore, the following result shows that every isolated invariant set *S* does indeed admit at least one index pair.

#### Proposition 5.3

Let *S* be an isolated invariant set. Then $\left( {\text {cl}}S, {\text {mo}}S\right)$ is a saturated index pair for *S*.

#### Proof

To prove (IP1) assume that $x\in {\text {mo}}S$ and $y\in \Pi _\mathcal {V}(x)\cap {\text {cl}}S$. Since *S* is $\mathcal {V}$-compatible we have $[x]_\mathcal {V}\cap S=\varnothing$. Therefore, $[x]_\mathcal {V}\cap {\text {cl}}S\subset {\text {cl}}S\setminus S={\text {mo}}S$. Clearly, due to Propositions 3.3 and 4.12, ${\text {cl}}x\subset {\text {mo}}S\subset {\text {cl}}S$. Hence,

$$\begin{aligned} y\in \Pi _\mathcal {V}(x)\cap {\text {cl}}S = ([x]_\mathcal {V}\cup {\text {cl}}x)\cap {\text {cl}}S = ([x]_\mathcal {V}\cap {\text {cl}}S) \cup ({\text {cl}}x \cap {\text {cl}}S) \subset {\text {mo}}S. \end{aligned}$$

To see (IP2) note that by Proposition 4.10 the set *S* is $\mathcal {V}$-compatible and

$$\begin{aligned} \Pi _\mathcal {V}(S)=\bigcup _{x\in S} {\text {cl}}x\cup [x]_\mathcal {V}= \bigcup _{x\in S} {\text {cl}}x\cup S = {\text {cl}}S. \end{aligned}$$

Thus, if $x\in S$, then $\Pi _\mathcal {V}(x) \setminus {\text {cl}}S = \varnothing$. Therefore, $\Pi _\mathcal {V}(x) \setminus {\text {cl}}S \ne \varnothing$ for $x\in P_1={\text {cl}}S$ implies $x\in {\text {cl}}S\setminus S={\text {mo}}S$.

Finally, directly from the definition of mouth we have ${\text {cl}}S \setminus {\text {mo}}S=S$, which proves (IP3), as well as the fact that $({\text {cl}}S,{\text {mo}}S)$ is saturated. $\square$

We write $P\subset Q$ for index pairs *P*, *Q* meaning $P_i\subset Q_i$ for $i=1,2$. We say that index pairs *P*, *Q* of *S* are *semi-equal* if $P\subset Q$ and either $P_1=Q_1$ or $P_2=Q_2$. For semi-equal pairs *P*, *Q*, we let

$$\begin{aligned} A(P,Q):={\left\{ \begin{array}{ll} Q_1\setminus P_1 &{} \text { if }P_2=Q_2,\\ Q_2\setminus P_2 &{} \text { if }P_1=Q_1. \end{array}\right. } \end{aligned}$$

**Fig. 10:**
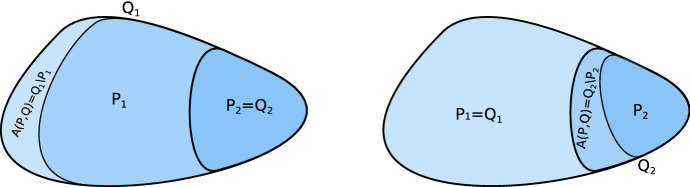
Schematic depiction of the two cases of a set *A*(*P*, *Q*)

#### Proposition 5.4

Let *P* and *Q* be semi-equal index pairs for *S*. Then there is no essential solution in the set *A*(*P*, *Q*).

#### Proof

First note that the definition of *A*(*P*, *Q*) implies either

$$\begin{aligned} A(P,Q) = Q_1\setminus P_1 \subset Q_1\setminus P_2 = Q_1\setminus Q_2 \quad \text{ and }\quad A(P,Q) \cap (P_1 \setminus P_2) = \varnothing , \end{aligned}$$

or

$$\begin{aligned} A(P,Q) = Q_2\setminus P_2 \subset Q_1\setminus P_2 = P_1\setminus P_2 \quad \text{ and }\quad A(P,Q) \cap (Q_1 \setminus Q_2) = \varnothing . \end{aligned}$$

Therefore, by (IP3) and the first inclusions in the above two statements we get ${\text {Inv}}A(P,Q)\subset S$. Yet, the second identities above clearly show that *A*(*P*, *Q*) is disjoint from *S*. Thus, ${\text {Inv}}A(P,Q) = \varnothing$, and by the definition of the invariant part (see (7)) there is no essential solution in *A*(*P*, *Q*). $\square$

#### Lemma 5.5

Assume *S* is an isolated invariant set. Let *P* and *Q* be saturated index pairs for *S*.

Then $$H(P_1,P_2)\cong H(Q_1,Q_2)$$.

#### Proof

By the definition of a saturated index pair $Q_1\setminus Q_2 = S = P_1\setminus P_2$. Hence, using Theorem 3.11(i) we get $H(P_1,P_2)\cong H(Q_1,Q_2)$. $\square$

#### Proposition 5.6

Assume *S* is an isolated invariant set. Let *P* be an index pair for *S*. Then the set $P_1 \setminus P_2$ is $\mathcal {V}$-compatible and locally closed.

#### Proof

Assume that $P_1 \setminus P_2$ is not $\mathcal {V}$-compatible. This means that for some $x \in P_1\setminus P_2$ there exists a $y\in [x]_\mathcal {V}\setminus (P_1 \setminus P_2)$. Then $y\in P_2$ or $y\not \in P_1$. Consider the case $y\in P_2$. Since $[x]_\mathcal {V}=[y]_\mathcal {V}$, we have $x\in \Pi _\mathcal {V}(y)$. It follows from (IP1) that $x\in P_2$, a contradiction. Consider now the case $y\not \in P_1$. Then from (IP2) one obtains $x\in P_2$, which is again a contradiction. Together, these cases imply that $P_1\setminus P_2$ is $\mathcal {V}$-compatible.

Finally, the local closedness of $P_1\setminus P_2$ follows immediately from Proposition 3.3(iii). $\square$

#### Proposition 5.7

Assume *S* is an isolated invariant set. Let $P \subset Q$ be semi-equal index pairs for *S*. Then *A*(*P*, *Q*) is $\mathcal {V}$-compatible and locally closed.

#### Proof

First note that our assumptions give $P_2,Q_2\subset P_1$ and $P_2,Q_2\subset Q_1$. If $P_2=Q_2$, then

$$\begin{aligned} A(P,Q) =Q_1\setminus P_1 = (Q_1\setminus P_2)\setminus (P_1\setminus P_2) = (Q_1\setminus Q_2)\setminus (P_1\setminus P_2). \end{aligned}$$

If $$P_1=Q_1$$, then

$$\begin{aligned} A(P,Q)&= Q_2\setminus P_2 = Q_2\cap P_2^c = (P_1\cap P_2^c)\cap Q_2\\&=(P_1\cap P_2^c)\cap (Q_1\cap Q_2^c)^c = (P_1\setminus P_2)\setminus (Q_1\setminus Q_2), \end{aligned}$$

where the superscript *c* denotes the set complement in *X*. Thus, by Proposition 5.6, in both cases, *A*(*P*, *Q*) may be represented as a difference of $\mathcal {V}$-compatible sets. Therefore, it is also $\mathcal {V}$-compatible.

The local closedness of *A*(*P*, *Q*) follows from Proposition 3.3. $\square$

#### Lemma 5.8

Let *A* be a $\mathcal {V}$-compatible, locally closed subset of *X* such that there is no essential solution in *A*. Then $H({\text {cl}}A, {\text {mo}}A)=0$.

#### Proof

Let $\mathcal {A}:=\{V\in \mathcal {V}\mid V\subset A\}$. Since *A* is $\mathcal {V}$-compatible, we have $A=\bigcup \mathcal {A}$. Let $\lesssim _\mathcal {A}$ denote the transitive closure of the relation $\preceq _\mathcal {A}$ in $\mathcal {A}$ given for $V,W\in \mathcal {A}$ by

8 $$\begin{aligned} V\preceq _\mathcal {A}W\ \Leftrightarrow \ V\cap {\text {cl}}W\ne \varnothing . \end{aligned}$$

We claim that $\lesssim _\mathcal {A}$ is a partial order in $\mathcal {A}$. Clearly, $\lesssim _\mathcal {A}$ is reflective and transitive. Hence, we only need to prove that $\lesssim _\mathcal {A}$ is antisymmetric. To verify this, suppose the contrary. Then there exists a cycle $V_n\preceq _\mathcal {A}V_{n-1}\preceq _\mathcal {A}\dots \preceq _\mathcal {A}V_0=V_n$ with $n>1$ and $V_i\ne V_j$ for $i\ne j$ and $i,j\in \{1,2,\dots n\}$. Since $V_i\cap {\text {cl}}V_{i-1}\ne \varnothing$ we can choose $v_i\in V_i\cap {\text {cl}}V_{i-1}$ and $v_{i-1}'\in V_{i-1}$ such that $v_i\in {\text {cl}}v_{i-1}'$. Then $v_i\in \Pi _\mathcal {V}(v_{i-1}')$ and $v_{i-1}'\in \Pi _\mathcal {V}(v_{i-1})$. Thus, we can construct an essential solution

$$\begin{aligned} \dotsc \cdot v_n'\cdot v_1\cdot v_1'\cdot v_2\cdot v_2'\cdot \dotsc \cdot v_{n-1}'\cdot v_n\cdot v_n'\cdot v_1\cdot \dotsc . \end{aligned}$$

This contradicts our assumption and proves that $\lesssim _\mathcal {A}$ is a partial order.

Moreover, since a constant solution in a critical multivector is essential, all multivectors in $\mathcal {A}$ have to be regular. Thus,

9 $$\begin{aligned} H({\text {cl}}V, {\text {mo}}V) = 0 \quad \text {for every}\quad V\in \mathcal {A}. \end{aligned}$$

Since $\lesssim _\mathcal {A}$ is a partial order, we may assume that $\mathcal {A}=\{V_i\}_{i=1}^m$ where the numbering of $V_i$ extends the partial order $\lesssim _\mathcal {A}$ to a linear order $\le _\mathcal {A}$, that is,

$$\begin{aligned} V_1 \le _\mathcal {A}V_2 \le _\mathcal {A}\dots \le _\mathcal {A}V_m. \end{aligned}$$

We claim that

10 $$\begin{aligned} i<j\quad \Rightarrow \quad {\text {cl}}V_i\setminus V_j={\text {cl}}V_i. \end{aligned}$$

Indeed, if this is not satisfied, then $V_j\cap {\text {cl}}V_i\ne \varnothing$ which, by the definition (8) of $\preceq _\mathcal {A}$ gives $V_j\preceq _\mathcal {A}V_i$ as well as $V_j\lesssim _\mathcal {A}V_i$, and therefore $j\le i$, a contradiction. For $k\in \{0,1,\dots m\}$ define set $W_k:=\bigcup _{j=1}^kV_j$. Then $W_0=\varnothing$ and $W_m=A$. Now fix a $k\in \{0,1,\dots m\}$. Observe that by (10) we have

$$\begin{aligned} {\text {cl}}W_k\setminus A = \bigcup _{j=1}^k{\text {cl}}V_j\setminus \bigcup _{j=1}^m V_j = \bigcup _{j=1}^k{\text {cl}}V_j\setminus \bigcup _{j=1}^k V_j = {\text {cl}}W_k\setminus W_k = {\text {mo}}W_k. \end{aligned}$$

Therefore,

$$\begin{aligned} {\text {mo}}W_k = {\text {cl}}W_k\setminus A\subset {\text {cl}}A\setminus A={\text {mo}}A. \end{aligned}$$

It follows that $W_k\cup {\text {mo}}A = {\text {cl}}W_k\cup {\text {mo}}A$. Hence, the set $Z_k:=W_k\cup {\text {mo}}A$ is closed. For $k>0$ we have

$$\begin{aligned} Z_k\setminus Z_{k-1} = W_k\setminus W_{k-1}\setminus {\text {mo}}A = V_k\cap A = V_k = {\text {cl}}V_k\setminus {\text {mo}}V_k. \end{aligned}$$

Hence, we get from Theorem 3.11(i) and (9)

$$\begin{aligned} H(Z_k,Z_{k-1}) = H({\text {cl}}V_k,{\text {mo}}V_k)=0. \end{aligned}$$

Now it follows from the exact sequence of the triple $(Z_{k-1}, Z_k, {\text {cl}}A)$ that

$$\begin{aligned} H({\text {cl}}A, Z_k)\cong H({\text {cl}}A,Z_{k-1}). \end{aligned}$$

Note that $Z_0=W_0\cup {\text {mo}}A = {\text {mo}}A$ and $Z_m=W_m\cup {\text {mo}}A=A\cup {\text {mo}}A={\text {cl}}A$. Therefore, we finally obtain

$$\begin{aligned} H({\text {cl}}A,{\text {mo}}A)=H({\text {cl}}A, Z_0)\cong H({\text {cl}}A,Z_m) = H({\text {cl}}A, {\text {cl}}A) = 0, \end{aligned}$$

which completes the proof of the lemma. $\square$

#### Lemma 5.9

Let $P \subset Q$ be semi-equal index pairs of an isolated invariant set *S*. If $P_1 = Q_1$, then $H(Q_2, P_2) = 0$, and analogously, if $P_2 = Q_2$, then $H(Q_1, P_1) = 0$.

#### Proof

By Theorem 5.7 the set *A*(*P*, *Q*) is locally closed and $\mathcal {V}$-compatible. Hence, the conclusion follows from Proposition 5.4 and Lemma 5.8. $\square$

#### Lemma 5.10

Let $P\subset Q$ be semi-equal index pairs of an isolated invariant set *S*. Then $H(P_1, P_2)\cong H(Q_1, Q_2)$.

#### Proof

Assume $P_2 = Q_2$. We get from Lemma 5.9 that $H(Q_1,P_1)=0$. Using Theorem 3.11(ii) for the triple $P_2\subset P_1\subset Q_1$ then implies

$$\begin{aligned} H(P_1,P_2) \cong H(Q_1, P_2) = H(Q_1, Q_2). \end{aligned}$$

Similarly, if $P_1 = Q_1$ we consider the triple $P_2\subset Q_2\subset Q_1$ and obtain

$$\begin{aligned} H(P_1, P_2)=H(Q_1, P_2)\cong H(Q_1, Q_2). \end{aligned}$$

$$\square $$

In order to show that two arbitrary index pairs carry the same homological information, we need to construct auxiliary, intermediate index pairs. To this end, we define the *push-forward* and the *pull-back* of a set *A* in *B*.

11 $$\begin{aligned} \pi _\mathcal {V}^+(A,B)&:= \{ x \in B \mid \ \exists _{\varphi \in {\text {Path}}_\mathcal {V}(B)}\ \varphi ^{\sqsubset } \in A,\ \varphi ^{\sqsupset } = x \}, \end{aligned}$$

12 $$\begin{aligned} \pi _\mathcal {V}^-(A,B)&:= \{ x \in B \mid \ \exists _{\varphi \in {\text {Path}}_\mathcal {V}(B)}\ \varphi ^{\sqsubset } = x,\ \varphi ^{\sqsupset } \in A\} \end{aligned}$$

#### Proposition 5.11

Let $A\subset X$ then $\pi ^+_\mathcal {V}(A,X)$ ($\pi ^-_\mathcal {V}(A,X)$) is closed (open) and $\mathcal {V}$-compatible.

#### Proof

Let $x\in \pi ^+_\mathcal {V}(A,X)$ be arbitrary. Then there exists a point $a\in A$ and a $\varphi \in {\text {Path}}_\mathcal {V}(a,x,X)$. For any $y\in [x]_\mathcal {V}$ the concatenation $\varphi \cdot y$ is also a path. Thus, $\pi ^+_\mathcal {V}(A,X)$ is $\mathcal {V}$-compatible.

To show closedness, take an $x\in \pi ^+_\mathcal {V}(A,X)$ and $y\in {\text {cl}}x$. By (11) there exists an $a\in A$ and a $\varphi \in {\text {Path}}_\mathcal {V}(a, x, X)$. Then the path $\varphi \cdot y$ is a path from *A* to *y*, implying that $y\in \pi ^+_\mathcal {V}(A,X)$. Since *X* is finite one obtains

$$\begin{aligned} {\text {cl}}\pi _\mathcal {V}^+(A,X)= \bigcup _{x\in \Pi _\mathcal {V}^+(A,X)}{\text {cl}}x = \pi _\mathcal {V}^+(A,X), \end{aligned}$$

and therefore $\pi _\mathcal {V}^+(A,X)$ is closed. The proof for $\pi _\mathcal {V}^-(A,X)$ is symmetric. $\square$

Let *P* be an index pair for *S*. Define the set $\hat{P} \subset P_1$ of all points $x\in P_1$ for which there exists no path in $P_1$ which starts in *x* and ends in *S*, that is,

13 $$\begin{aligned} \hat{P} := \{ x\in P_1\ |\ \pi ^+_\mathcal {V}(x,P_1)\cap S=\varnothing \} . \end{aligned}$$

#### Proposition 5.12

If *P* is an index pair for an isolated invariant set *S*, then $S\cap \hat{P}=\varnothing$ and $P_2\subset \hat{P}$.

#### Proof

The first assertion is obvious. In order to see the second take an $x\in P_2$ and suppose that $x\not \in \hat{P}$. This means that there exists a path $\varphi$ in $P_1$ such that $\varphi ^{\sqsubset }=x$ and $\varphi ^{\sqsupset }\in S$. The condition (IP1) of Definition 5.1 implies ${\text {im}}\varphi \in P_2$. Therefore, $\varphi ^{\sqsupset }\in P_2$ and $P_2\cap S\ne \varnothing$ which contradicts $S\subset P_1\setminus P_2$. $\square$

#### Proposition 5.13

If *P* is an index pair for an isolated invariant set *S*, then ${\text {mo}}S\subset \hat{P}$. Moreover, $\Pi _\mathcal {V}(S)\subset S\cup \hat{P}$.

#### Proof

To prove that ${\text {mo}}S\subset \hat{P}$ assume the contrary. Then there exists an $x\in {\text {mo}}S$, such that $\pi ^+_\mathcal {V}(x,P_1)\cap S\ne \varnothing$. It follows that there exists a path $\varphi$ in $P_1$ from *x* to *S*. Since $x\in {\text {mo}}S\subset {\text {cl}}S$, we can take a $y\in S$ such that $x\in {\text {cl}}y\subset \Pi _\mathcal {V}(y)$. It follows that $\psi :=y\cdot \varphi$ is a path in $P_1$ through *x* with endpoints in *S*. Since, by Proposition 5.2, $P_1$ isolates *S*, we get $x\in S$, a contradiction. Finally, by $\mathcal {V}$-compatibility of *S* guaranteed by Proposition 4.10, we have the inclusion $\Pi _\mathcal {V}(S)={\text {cl}}S \subset S\cup {\text {mo}}S\cup \hat{P}= S\cup \hat{P}$, which proves the remaining assertion. $\square$

#### Proposition 5.14

Let *P* be an index pair for an isolated invariant set *S*. Then the sets $\hat{P}$ and $\hat{P}\cup S$ are closed.

#### Proof

Let $x\in \hat{P}$ and let $y\in {\text {cl}}x$. Then $y\in \Pi _\mathcal {V}(x)$. Moreover, $y\in P_1$, because $P_1$ is closed. Clearly, if $\varphi \in {\text {Path}}_\mathcal {V}(y,P_1)$, then $x\cdot \varphi \in {\text {Path}}_\mathcal {V}(x,P_1)$. Therefore $\pi ^+_\mathcal {V}(y,P_1)\subset \pi ^+_\mathcal {V}(x,P_1)$. Since, by (13), the latter set is disjoint from *S*, so is the former one. Therefore, $y\in \hat{P}$. It follows that $\hat{P}$ is closed.

Proposition 5.13 implies that ${\text {cl}}(S\cup \hat{P})={\text {cl}}S\cup \hat{P}=S\cup {\text {mo}}{S}\cup \hat{P}=S\cup \hat{P}$, which proves the closedness of $S\cup \hat{P}$. $\square$

#### Lemma 5.15

If *P* is an index pair for an isolated invariant set *S*, then $$P^*:=(S\cup \hat{P}, P_2)$$ is an index pair for *S* and $$P^{**}:=(S\cup \hat{P}, \hat{P})$$ is a saturated index pair for *S*.

#### Proof

First consider $P^*$. By Proposition 5.14 set $P^*_1=S\cup \hat{P}$ is closed. By Proposition 5.12 we have $P_2\subset \hat{P}\subset S\cup \hat{P}$.

Let $x\in P_2^*=P_2$ and let $y\in \Pi _\mathcal {V}(x)\cap P_1^*$. Then $y\in \Pi _\mathcal {V}(x)\cap P_1$. It follows from (IP1) for *P* that $y\in P_2$. Thus, (IP1) is satisfied for $P^*$.

Now, let $x\in P_1^*=S\cup \hat{P}$ and suppose that there is a $y\in \Pi _\mathcal {V}(x)\setminus P_1^*\ne \varnothing$. We have $x\not \in S$, because otherwise $\Pi _\mathcal {V}(x)\subset {\text {mo}}S\cup S={\text {cl}}S\subset {\text {cl}}(S\cup \hat{P})$ and then Proposition 5.14 implies $\Pi _\mathcal {V}(x)\subset S\cup \hat{P}\subset P_1^*$ which contradicts $\Pi _\mathcal {V}(x)\setminus P_1^* \ne \varnothing$. Hence, $x\in \hat{P}$. We have $y\not \in P_1$ because otherwise $y\in \pi _\mathcal {V}^+(x,P_1)\subset \hat{P}\subset P_1^*$, a contradiction. Thus $\Pi _\mathcal {V}(x)\setminus P_1\ne \varnothing$. Since $x\in P_1^*\subset P_1$, by (IP2) for *P* we get $x\in P_2=P_2^*$. This proves (IP2) for $P^*$.

Clearly, $P_1^*\setminus P_2^* = P_1^*\setminus P_2\subset P_1\setminus P_2$, and therefore we have the inclusion ${\text {Inv}}\left( P^*_1\setminus P^*_2 \right) \subset {\text {Inv}}\left( P_1\setminus P_2 \right) =S$. To verify the opposite inclusion, let $x\in S$ be arbitrary. Since *S* is an invariant set, there exists an essential solution $\varphi \in {\text {eSol}}(x, S)$. We have ${\text {im}}\varphi \subset S\subset (\hat{P}\cup S)\setminus P_2 =P^*_1\setminus P^*_2,$ because $P_2\cap S=\varnothing$. Consequently, $x\in {\text {Inv}}(P^*_1\setminus P^*_2)$ and $S={\text {Inv}}(P^*_1\setminus P^*_2)$. Hence, $P^*$ also satisfies (IP3), which completes the proof that $P^*$ is an index pair for *S*.

Consider now the second pair $$P^{**}$$. Let $$x\in P_2^{**}=\hat{P}$$ be arbitrary and choose $$y\in \Pi _\mathcal {V}(x)\cap P_1^{**}=\Pi _\mathcal {V}(x)\cap (\hat{P}\cup S)$$. Since $$x\in \hat{P}$$ we get from (13) that $$\Pi _\mathcal {V}(x)\cap S=\varnothing $$. Thus, $$y\in \Pi _\mathcal {V}(x)\cap \hat{P}\subset \hat{P}=P_2^{**}$$. This proves (IP1) for the pair $$P^{**}$$.

To see (IP2) take an $$x\in P_1^{**}=\hat{P}\cup S$$ and assume $$\Pi _\mathcal {V}(x)\setminus P_1^{**}\ne \varnothing $$. We cannot have $$x\in S$$, because then $$\Pi _\mathcal {V}(x)\subset \Pi _\mathcal {V}(S)$$ and Proposition 5.13 implies $$\Pi _\mathcal {V}(x)\subset S\cup \hat{P}=P_1^{**}$$, a contradiction. Hence, $$x\in \hat{P}=P_2^{**}$$ which proves (IP2) for $$P^{**}$$.

Finally, we clearly have $S\cap \hat{P}=\varnothing$. Therefore, $(S\cup \hat{P})\setminus \hat{P}= S$ and

$$\begin{aligned} {\text {Inv}}(P_1^{**}\setminus P_2^{**}) = {\text {Inv}}((S\cup \hat{P})\setminus \hat{P}) = {\text {Inv}}{S}= S. \end{aligned}$$

This proves that $$P^{**}$$ satisfies (IP3) and that it is saturated. $$\square $$

#### Theorem 5.16

Let *P* and *Q* be two index pairs for an invariant set *S*. Then $H(P_1, P_2)\cong H(Q_1, Q_2)$.

#### Proof

It follows from Lemma 5.15 that $$P^{*}\subset P$$ as well as $$P^*\subset P^{**}$$ are semi-equal index pairs. Hence, we get from Lemma 5.10 that

$$\begin{aligned} H(P_1,P_2)&\cong H(P_1^{*},P_2^{*})\cong H(P_1^{**},P_2^{**}). \end{aligned}$$

Similarly, one obtains

$$\begin{aligned} H(Q_1,Q_2)&\cong H(Q_1^{*},Q_2^{*})\cong H(Q_1^{**},Q_2^{**}). \end{aligned}$$

Since both pairs $$P^{**}$$ and $$Q^{**}$$ are saturated, it follows from Lemma 5.5 that $$H(P_1^{**},P_2^{**}) \cong H(Q_1^{**},Q_2^{**}).$$ Therefore, $$H(P_1,P_2) \cong H(Q_1,Q_2)$$. $$\square $$

### Conley index

We define the *homology Conley index* of an isolated invariant set *S* as $H(P_1,P_2)$ where $(P_1, P_2)$ is an index pair for *S*. We denote the homology Conley index of *S* by ${\text {Con}}(S)$. Proposition 5.3 and Theorem 5.16 guarantee that the homology Conley index is well-defined.

Given a locally closed set $A\subset X$ we define its *ith Betti number* $\beta _i(A)$ and *Poincaré polynomial* $p_A(t)$, respectively, as the *i*th Betti number and the Poincaré polynomial of the pair $({\text {cl}}A,{\text {mo}}A)$, that is, $\beta _i(A):=\beta _i({\text {cl}}A, {\text {mo}}A)$ and $p_A(t):=p_{{\text {cl}}A, {\text {mo}}A}(t)$ (see (4)).

The theorem used in the following Proposition originally comes from Rybakowski and Zehnder (1985), but we use its more general version that was stated in Mrozek (2017).

#### Proposition 5.17

If $(P_1,P_2)$ is an index pair for an isolated invariant set *S*, then

14 $$\begin{aligned} p_S(t) + p_{P_2}(t) = p_{P_1}(t) + (1+t)q(t), \end{aligned}$$

where *q*(*t*) is a polynomial with non-negative coefficients. Moreover, if

$$\begin{aligned} H(P_1) = H(P_2) \oplus H({\text {cl}}S, {\text {mo}}S) \end{aligned}$$

then $$q(t)=0$$.

#### Proof

An index pair $(P_1, P_2)$ induces a long exact sequence of homology modules

15 $$\begin{aligned} \dotsc \rightarrow H_n(P_2) \rightarrow H_n(P_1) \rightarrow H_n(P_1, P_2) \rightarrow H_{n-1}(P_2) \rightarrow \dotsc . \end{aligned}$$

By Proposition 5.3 and Theorem 5.16 we have $H(P_1, P_2)\cong H({\text {cl}}S, {\text {mo}}S)$. Thus, we can replace (15) with

$$\begin{aligned} \dotsc \rightarrow H_n(P_2) \rightarrow H_n(P_1) \rightarrow H_n({\text {cl}}S, {\text {mo}}S) \rightarrow H_{n-1}(P_2) \rightarrow \dotsc . \end{aligned}$$

In view of [Mrozek 2017, Theorem 4.6] we further get

$$\begin{aligned} p_{S}(t) + p_{P_2}(t) = p_{P_1}(t) + (1+t)q(t). \end{aligned}$$

for some polynomial *q* with non-negative coefficients. The second assertion follows directly from the second part of [Mrozek 2017, Theorem 4.6] (see also Rybakowski and Zehnder 1985). $\square$

We say that an isolated invariant set *S* *decomposes* into the isolated invariant sets $S'$ and $S''$ if ${\text {cl}}S'\cap S''=\varnothing$, $S'\cap {\text {cl}}S''=\varnothing$, as well as $S=S'\cup S''$.

#### Proposition 5.18

Assume an isolated invariant set *S* decomposes into the isolated invariant sets $S'$ and $S''$. Then ${\text {Sol}}(S)={\text {Sol}}(S')\cup {\text {Sol}}(S'')$.

#### Proof

The inclusion ${\text {Sol}}(S')\cup {\text {Sol}}(S'')\subset {\text {Sol}}(S)$ is trivial. To see the opposite inclusion, let $\varphi \in {\text {Sol}}(S)$. We have to prove that ${\text {im}}\varphi \subset S'$ or ${\text {im}}\varphi \subset S''$. If this were not the case, then without loss of generality we can assume that there exists a $j\in {\mathbb {Z}}$ such that both $\varphi (j)\in S'$ and $\varphi (j+1)\in S''$ are satisfied. This immediately implies $\varphi (j+1)\in {\text {cl}}\varphi (j)\cup [\varphi (j)]_\mathcal {V}$. We have $\varphi (j+1)\not \in [\varphi (j)]_\mathcal {V}$, because otherwise the $\mathcal {V}$-compatibility of $S'$ (see Propostition 4.10) implies $\varphi (j+1)\in S'$ and, in consequence, $S'\cap S''\ne \varnothing$, a contradiction. Hence, $\varphi (j+1)\in {\text {cl}}\varphi (j)\subset {\text {cl}}S'$ which yields ${\text {cl}}S'\cap S''\ne \varnothing$, another contradiction, proving that $\varphi \in {\text {Sol}}(S')\cup {\text {Sol}}(S'')$. $\square$

#### Theorem 5.19

Assume an isolated invariant set *S* decomposes into the isolated invariant sets $S'$ and $S''$. Then we have

$$\begin{aligned} {\text {Con}}(S)={\text {Con}}(S')\oplus {\text {Con}}(S''). \end{aligned}$$

#### Proof

In view of Proposition 5.3, the two pairs $P=({\text {cl}}S',{\text {mo}}S')$ and $Q=({\text {cl}}S'', {\text {mo}}S'')$ are saturated index pairs for $S'$ and $S''$, respectively. Consider the following exact sequence given by Theorem 3.11(iii):

16 $$\begin{aligned} \begin{aligned} \dotsc \rightarrow&H_n(P_1\cap Q_1, P_2\cap Q_2) \rightarrow H_n(P_1, P_2)\oplus H_n(Q_1, Q_2)\\ \rightarrow&H_n(P_1\cup Q_1, P_2\cup Q_2) \rightarrow H_{n-1}(P_1\cap Q_1, P_2\cap Q_2) \rightarrow \dotsc . \end{aligned} \end{aligned}$$

Note that $S'\cap Q_2\subset S'\cap {\text {cl}}S''=\varnothing$ and similarly $S''\cap P_2=\varnothing$. Since both *P* and *Q* are saturated and $S'\cap S''=\varnothing$ we get

$$\begin{aligned} P_1\cap Q_1&= (S'\cup P_2)\cap (S''\cup Q_2)\\&=(S'\cap S'')\cup (S'\cap Q_2)\cup (P_2\cap S'')\cup (P_2\cap Q_2) =P_2\cap Q_2. \end{aligned}$$

Thus, $H(P_1\cap Q_1, P_2\cap Q_2)=0$, which together with the exact sequence (16) implies

17 $$\begin{aligned} H_*(P_1\cup Q_1,P_2\cup Q_2)\cong H_*(P_1,P_2)\oplus H_*(Q_1,Q_2). \end{aligned}$$

Notice further that $S'\cap {\text {cl}}S''=\varnothing$ implies $S'\setminus Q_2=S'$. Similarly $S''\setminus P_2=S''$. Therefore, one obtains the identity

$$\begin{aligned} (P_1\setminus P_2\setminus Q_2)\cup (Q_1\setminus Q_2\setminus P_2) = (S'\setminus Q_2)\cup (S''\setminus P_2) = S'\cup S'' = S. \end{aligned}$$

Hence, by Theorem 3.11(i),

18 $$\begin{aligned} H({\text {cl}}S, {\text {mo}}S)\cong H(P_1\cup Q_1, P_2\cup Q_2). \end{aligned}$$

Finally, from (17) and (18) we get

$$\begin{aligned} {\text {Con}}(S)&= H({\text {cl}}S, {\text {mo}}S)\cong H(P_1\cup Q_1, P_2\cup Q_2) \\&\cong H(P_1, P_2)\oplus H(Q_1, Q_2)= {\text {Con}}(S')\oplus {\text {Con}}(S''), \end{aligned}$$

which completes the proof of the theorem. $\square$

## Attractors, repellers and limit sets

In the rest of the paper we assume that the finite topological space *X* is invariant with respect to a given combinatorial multivector field $\mathcal {V}$ on *X*. We need the invariance assumption to guarantee the existence of an essential solution through every point in *X*. This assumption is not very restrictive, because if *X* is not invariant, then we can replace the space *X* by its invariant part ${\text {Inv}}X$ and the multivector field $\mathcal {V}$ by its restriction $\mathcal {V}_{{\text {Inv}}X}$ (see Propositions 4.2 and 4.9).

### Attractors, repellers and indecomposable sets

We say that an invariant set $A\subset X$ is an *attractor* if $\Pi _\mathcal {V}(A) = A$. In addition, an invariant set $R\subset X$ is a *repeller* if $\Pi ^{-1}_\mathcal {V}(R) = R$.

The following proposition shows that we can also express the concepts of attractor and repeller in terms of push-forward and pull-back.

#### Proposition 6.1

Let *A* be an invariant set. Then *A* is an attractor (a repeller) in *X* if and only if $\pi _\mathcal {V}^+(A,X)=A$ ($\pi _\mathcal {V}^-(A,X)=A$).

#### Proof

Let *A* be an attractor. The inclusion $S\subset \pi _\mathcal {V}^+(S,X)$ is true for an arbitrary set. Suppose that there exists a $y\in \pi _\mathcal {V}^+(A,X)\setminus A$. Then by (11) we can find an $x\in A$ and $\varphi \in {\text {Path}}_\mathcal {V}(x,y,X)$. This implies that there exists a $k\in {\mathbb {Z}}$ such that $\varphi (k)\in A$ and $\varphi (k+1)\not \in A$. But $\varphi (k+1)\in \Pi _\mathcal {V}(\varphi (k))\subset \Pi _\mathcal {V}(A)=A$, a contradiction. Therefore, $\pi _\mathcal {V}^+(A,X)=A$.

Now assume that $\pi _\mathcal {V}^+(A,X)=A$. Again, by (11), we get $A=\pi _\mathcal {V}^+(A,X)=\Pi _\mathcal {V}(\pi _\mathcal {V}^+(A,X))=\Pi _\mathcal {V}(A)$.

The proof for a repeller is analogous. $\square$

#### Theorem 6.2

The following conditions are equivalent:

1. *A* is an attractor,
2. *A* is closed, $\mathcal {V}$-compatible, and invariant,
3. *A* is a closed isolated invariant set.

#### Proof

Let *A* be an attractor. It follows immediately from Propositions 6.1 and 5.11 that condition *(1)* implies condition *(2)*.

Moreover, Proposition 4.13 shows that *(2)* implies *(3)*. Finally, suppose that *(3)* holds. By Proposition 4.10 set *A* is $\mathcal {V}$-compatible. It is also closed. Therefore, we have

$$\begin{aligned} \Pi _\mathcal {V}(A) = \bigcup _{x\in A}{\text {cl}}x\cup [x]_{\mathcal {V}} = \bigcup _{x\in A}{\text {cl}}x \cup \bigcup _{x\in A}[x]_{\mathcal {V}} = {\text {cl}}A\cup A= A, \end{aligned}$$

which proves that *A* is an attractor. $\square$

#### Theorem 6.3

The following conditions are equivalent:

1. *R* is a repeller,
2. *R* is open, $\mathcal {V}$-compatible, and invariant,
3. *R* is an open isolated invariant set.

#### Proof

Assume *R* is a repeller. It follows from Propositions 6.1 and 5.11 that condition *(1)* implies condition *(2)*, and Proposition 4.13 shows that *(2)* implies *(3)*. Finally, assume that condition *(3)* holds. Then *R* is $\mathcal {V}$-compatible by Proposition 4.10. The openness of *R* and Proposition 4.5 imply

$$\begin{aligned} \Pi ^{-1}_\mathcal {V}(R) = \bigcup _{x\in R}{\text {opn}}x\cup [x]_{\mathcal {V}} = \bigcup _{x\in R}{\text {opn}}x \cup \bigcup _{x\in R}[x]_{\mathcal {V}} = R, \end{aligned}$$

which proves that *R* is a repeller. $\square$

Let $\varphi$ be a full solution in *X*. We define the *ultimate backward* and *forward image* of $\varphi$ respectively by

$$\begin{aligned} {\text {uim}}^-{\varphi }&:=\bigcap _{t\in {\mathbb {Z}}^-}\varphi \left( (-\infty ,t] \right) ,\\ {\text {uim}}^+{\varphi }&:=\bigcap _{t\in {\mathbb {Z}}^+}\varphi \left( [t,+\infty ) \right) . \end{aligned}$$

Note that in a finite space a descending sequence of sets eventually must become constant. Therefore, we get the following result.

#### Proposition 6.4

There exists a $k\in {\mathbb {N}}$ such that ${\text {uim}}^-{\varphi } = \varphi ( (-\infty ,-k])$ and ${\text {uim}}^+{\varphi } = \varphi ( [k,+\infty ))$. In particular, the sets ${\text {uim}}^-{\varphi }$ and ${\text {uim}}^+{\varphi }$ are always non-empty.

#### Proposition 6.5

If $\varphi$ is a left-essential (a right-essential) solution, then we can find an essential solution $\psi$ such that ${\text {im}}\psi \subset {\text {uim}}^-\varphi$ (${\text {im}}\psi \subset {\text {uim}}^+\varphi$).

#### Proof

We only consider the case of a right-essential solution $\varphi$. By Proposition 6.4 there exists a $k\in {\mathbb {Z}}$ such that ${\text {uim}}^+\varphi =\varphi ([k,+\infty ))$. We consider two cases. If ${\text {uim}}^+\varphi$ passes through a critical multivector, then we can easily build a stationary essential solution. In the second case, we have at least two different multivectors $V,W\in \mathcal {V}$ such that $V\cap {\text {uim}}^+\varphi \ne \varnothing \ne W\cap {\text {uim}}^+\varphi$. Then there exist $t,s,u\in {\mathbb {Z}}$ with $k<t<s<u$ and $\varphi (t)\in V$, $\varphi (s)\in W$, and $\varphi (u)\in V$. But then the concatenation $\dots \cdot \varphi ([t,u])\cdot \varphi ([t,u])\cdot \dots$ is clearly essential. $\square$

#### Definition 6.6

We say that a non-empty invariant set $A\subset X$ is *Morse indecomposable*, or briefly *indecomposable*, if the only non-empty attractor in *A* is the entire set *A*.

#### Proposition 6.7

Let $A\subset X$ be a non-empty invariant set. Then *A* is indecomposable if and only if *A* is a strongly connected set in $G_\mathcal {V}$.

#### Proof

Let *A* be an indecomposable invariant set. Suppose it is not strongly connected. Then we can find points $x,y\in A$ such that ${\text {Path}}_\mathcal {V}(x,y,A)=\varnothing$. Define $A':={\text {Inv}}\pi _\mathcal {V}^+(x,A)$. Clearly, $y\not \in A'$. We will show that $A'$ is a non-empty attractor in *A*.

Let $z\in \pi _\mathcal {V}^+(x,A)$. Since *A* is invariant there exists $\varphi \in {\text {eSol}}_\mathcal {V}^+(z, A)$. By Proposition 6.5 we can construct an essential solution $\psi$ such that ${\text {im}}\psi \subset {\text {uim}}^+\varphi \subset \pi _\mathcal {V}^+(x,A)$. Thus, $A'$ is non-empty.

Now suppose that $\pi _\mathcal {V}^+(A',A)\ne A'$. Then there exists an $a\in \pi _\mathcal {V}^+(A',A)\setminus A'$. It follows from (11) that for every $b\in A'$ we have ${\text {Path}}_\mathcal {V}(a,b,A)=\varnothing$, since otherwise we could construct an essential solution through *a* which lies in $\pi _\mathcal {V}^+(x,A)$. Using exactly the same reasoning as above, one can further show that ${\text {Inv}}(\pi _\mathcal {V}^+(A',A)\setminus A')\ne \varnothing$. But since we clearly have the inclusion ${\text {Inv}}(\pi _\mathcal {V}^+(A',A)\setminus A')\subset {\text {Inv}}(\pi _\mathcal {V}^+(x,A))= A'$, this leads to a contradiction. Thus, Proposition 6.1 shows that the set $A'$ is indeed an attractor, which is non-empty and a proper subset of *A*. Since this contradicts the minimality of *A*, we therefore conclude that *A* is strongly connected.

Now assume conversely that *A* is strongly connected. It is clear that for any point $x\in A$ we get $\pi _\mathcal {V}^+(x,A)=A$. It follows by Proposition 6.1 that the only non-empty attractor in *A* is the entire set *A*. $\square$

The duality allows us to adapt the proof of Proposition 6.7 to get the following proposition.

#### Proposition 6.8

An invariant set *R* is an indecomposable invariant set if and only if the only non-empty repeller in *R* is the entire set *R*.

#### Proposition 6.9

Let $S\subset X$ be an indecomposable invariant set and let $A\subset X$ be an attractor (a repeller). If $A\cap S\ne \varnothing$ then $S\subset A$.

#### Proof

Let $x\in A\cap S$ and let $y\in S$. There exists $\varphi \in {\text {Path}}_\mathcal {V}(x,y,S)$. Now define $t=\min {\text {dom}}{\varphi }$ and $s=\max {\text {dom}}{\varphi }$. Clearly

$$\begin{aligned} \varphi (t+1)\in \Pi _\mathcal {V}(\varphi (t))=\Pi _\mathcal {V}(x)\subset \Pi _\mathcal {V}(A)=A. \end{aligned}$$

Now, by induction let $k\in \{t,t+1,\dots ,s-1\}$ and $\varphi (k)\in A$ then

$$\begin{aligned} \varphi (k+1)\in \Pi _\mathcal {V}(\varphi (k))\subset \Pi _\mathcal {V}(A)=A. \end{aligned}$$

Therefore, $y=\varphi (k+1)\in A$, and this implies $S\subset A$. $\square$

For a full solution $\varphi$ in *X*, define the sets

19 $$\begin{aligned}&\mathcal {V}^-(\varphi ) := \left\{ V\in \mathcal {V}\mid V\cap {\text {uim}}^-\varphi \ne \varnothing \right\} , \end{aligned}$$

20 $$\begin{aligned}&\mathcal {V}^+(\varphi ) := \left\{ V\in \mathcal {V}\mid V\cap {\text {uim}}^+\varphi \ne \varnothing \right\} . \end{aligned}$$

We refer to a multivector $V\in \mathcal {V}^-(\varphi )$ (respectively $\mathcal {V}^+(\varphi )$) as a *backward* (respectively *forward*) *ultimate multivector* of $\varphi$. The families $\mathcal {V}^-(\varphi )$ and $\mathcal {V}^+(\varphi )$ will be used in the sequel, in particular in the proof of the following theorem.

#### Theorem 6.10

Let $A\subset X$ be an attractor. Then $A^\star :={\text {Inv}}\left( X\setminus A\right)$ is a repeller in *X*, which is called the *dual repeller* of *A*. Conversely, if *R* is a repeller, then $R^\star :={\text {Inv}}\left( X\setminus R\right)$ is an attractor in *X*, called the *dual attractor* of *R*. Moreover, the dual repeller (or the dual attractor) is non-empty, unless we have $A=X$ (or $R=X$).

#### Proof

We will show that $A^\star$ is open. Let $x\in A^\star$ and let $y\in {\text {opn}}x$. Then one has $x\in {\text {cl}}y$ by Proposition 3.9. Since *A* is closed as an attractor (Proposition 6.2), we immediately get $y\not \in A$. The invariance of *X* lets us select a $\varphi \in {\text {eSol}}(y,X)$. Then ${\text {im}}\varphi ^-\cap A=\varnothing$, because otherwise there exists a $t\in {\mathbb {Z}}^-$ such that $\varphi (t)\in A$ and $\varphi (t+1)\not \in A$, which gives

$$\begin{aligned} \varphi (t+1)\in \Pi _\mathcal {V}(\varphi (t))\subset \Pi _\mathcal {V}(A)=A, \end{aligned}$$

a contradiction. Now, let $\psi \in {\text {eSol}}(x,A^\star )$. Clearly, $x\in {\text {cl}}y\subset \Pi _\mathcal {V}(y)$. Thus, $\varphi ^-\cdot \psi ^+\in {\text {eSol}}(y,X\setminus A)$. It follows that $y\in {\text {Inv}}(X\setminus A)=A^\star$ which proves that ${\text {opn}}A^\star \subset A^\star$. Therefore, the set $A^\star$ is open.

Since *A* is $\mathcal {V}$-compatible, also $X\setminus A$ is $\mathcal {V}$-compatible. Let $x\in A^\star$ and let $y\in [x]_\mathcal {V}$. Since $x\in A^\star \subset X\setminus A$, $\mathcal {V}$-compatibility of $X\setminus A$ implies $y\not \in A$. Select a $\varphi \in {\text {eSol}}(x,A^\star )$ Thus, $\varphi ^-\cdot y\cdot \varphi ^+$ is a well-defined essential solution in $X\setminus A$, that is, ${\text {eSol}}(y,X\setminus A)\ne \varnothing$. It follows that $y\in {\text {Inv}}(X\setminus A)=A^\star$. Hence, $A^\star$ is $\mathcal {V}$-compatible. Altogether, the set $A^\star$ is invariant, open and $\mathcal {V}$-compatible. Thus, by Theorem 6.3 it is a repeller.

Finally, we will show that $A^\star \ne \varnothing$ unless $A=X$. Suppose that $X\setminus A\ne \varnothing$, and let $x\in X\setminus A$. Since *X* is invariant, there exists a $\varphi \in {\text {eSol}}(x,X)$. As in the first part of the proof one can show that ${\text {im}}\varphi ^-\cap A=\varnothing$, that is, we have ${\text {im}}\varphi ^- \subset X \setminus A$. According to Proposition 6.5 there exists an essential solution $\psi$ such that ${\text {im}}\psi \subset {\text {uim}}^-\varphi \subset {\text {im}}\varphi ^- \subset X \setminus A$, and this immediately implies $A^\star = {\text {Inv}}\left( X\setminus A\right) \ne \varnothing$. $\square$

### Limit sets

We define the $\mathcal {V}$-*hull of a set* $A\subset X$ as the intersection of all $\mathcal {V}$-compatible, locally closed sets containing *A*, and denote it by $\langle A\rangle _\mathcal {V}$. As an immediate consequence of Proposition 3.4 and Proposition 4.3 we get the following result.

#### Proposition 6.11

For every $A\subset X$ its $\mathcal {V}$-hull is $\mathcal {V}$-compatible and locally closed.

We define the $\alpha$- *and* $\omega$-*limit sets of a full solution* $\varphi$ respectively by

$$\begin{aligned} \alpha (\varphi )&:= \left\langle {\text {uim}}^-\varphi \right\rangle _{\mathcal {V}},\\ \omega (\varphi )&:= \left\langle {\text {uim}}^+\varphi \right\rangle _{\mathcal {V}}. \end{aligned}$$

The following proposition is an immediate consequence of Proposition 3.6.

#### Proposition 6.12

Assume $\varphi$ is a full solution of $\mathcal {V}$ and $\varphi ^{{\text {op}}}$ is the associated dual solution of $\mathcal {V}^{{\text {op}}}$. Then $\alpha (\varphi ) = \omega (\varphi ^{{\text {op}}}) \quad \text {and}\quad \omega (\varphi ) = \alpha (\varphi ^{{\text {op}}})$.

#### Proposition 6.13

Let $\varphi$ be an essential solution. Then

$$\begin{aligned} \alpha (\varphi ) = \left\langle \bigcup \mathcal {V}^-(\varphi ) \right\rangle _{\mathcal {V}} \end{aligned}$$

and

$$\begin{aligned} \omega (\varphi ) = \left\langle \bigcup \mathcal {V}^+(\varphi ) \right\rangle _{\mathcal {V}}. \end{aligned}$$

#### Proof

Clearly

$$\begin{aligned} {\text {uim}}^-\varphi \subset \bigcup \left\{ V\in \mathcal {V}\mid V\cap {\text {uim}}^-\varphi \ne \varnothing \right\} = \bigcup \mathcal {V}^-(\varphi ) \end{aligned}$$

and therefore

$$\begin{aligned} \alpha (\varphi ) = \left\langle {\text {uim}}^-\varphi \right\rangle _\mathcal {V}\subset \left\langle \bigcup \mathcal {V}^-(\varphi ) \right\rangle _{\mathcal {V}}. \end{aligned}$$

Now let $x\in \bigcup \mathcal {V}^-(\varphi )$. Then there exists a $y\in [x]_{\mathcal {V}}$ such that $y\in {\text {uim}}^-\varphi$. Then $y\in \alpha (\varphi )$ and, since $\alpha (\varphi )$ is $\mathcal {V}$-compatible, $[y]_{\mathcal {V}}=[x]_{\mathcal {V}}\subset \alpha (\varphi )$. Thus, we have $\bigcup {\mathcal {V}^-({\varphi })}\subset \alpha (\varphi )$. Since $\alpha (\varphi )$ is locally closed and $\mathcal {V}$-compatible, the set $\alpha (\varphi )$ is a superset of the $\mathcal {V}$-hull of $\bigcup \alpha _\mathcal {V}(\varphi )$. Hence,

$$\begin{aligned} \left\langle \bigcup \mathcal {V}^-(\varphi ) \right\rangle _{\mathcal {V}} \subset \alpha (\varphi ). \end{aligned}$$

The proof for $\omega (\varphi )$ is analogous. $\square$

#### Lemma 6.14

Assume $\varphi :{\mathbb {Z}}\rightarrow X$ is a full solution of $\mathcal {V}$ and $\mathcal {V}^-(\varphi )$ (respectively $\mathcal {V}^+(\varphi )$) contains at least two different multivectors. Then for every $V\in \mathcal {V}$ such that $V\subset \alpha (\varphi )$ (respectively $V\subset \omega (\varphi )$) we have

21 $$\begin{aligned} \left( \Pi _\mathcal {V}(V)\setminus V \right) \cap \alpha (\varphi )\ne \varnothing \;\;\; \text {(respectively } \left( \Pi _\mathcal {V}(V)\setminus V \right) \cap \omega (\varphi )\ne \varnothing \text {)} \end{aligned}$$

and

22 $$\begin{aligned} \left( \Pi _\mathcal {V}^{-1}(V)\setminus V \right) \cap \alpha (\varphi )\ne \varnothing \;\;\; \text {(respectively } \left( \Pi _\mathcal {V}^{-1}(V)\setminus V \right) \cap \omega (\varphi )\ne \varnothing \text {).} \end{aligned}$$

#### Proof

Assume $V\in \mathcal {V}$ is such that $V\subset \alpha (\varphi )$. This happens if $V\in \mathcal {V}^-(\varphi )$, but might also happen for some $V\not \in \mathcal {V}^-(\varphi )$.

Assume first that $V\in \mathcal {V}^-(\varphi )$. Since there are at least two different multivectors in the set $\mathcal {V}^-(\varphi )$ there exists a strictly decreasing sequence $k:{\mathbb {N}}\rightarrow {\mathbb {Z}}^-$ such that $\varphi (k_n)\in V$ and $\varphi (k_n+1)\not \in V$. Since the set $\{\varphi (k_n+1)\mid n\in {\mathbb {N}}\}\subset X$ is finite, after taking a subsequence, if necessary, we may assume that $\varphi (k_n+1)=y\not \in V$. Let $W:=[y]_\mathcal {V}$. Then $W\ne V$ and $y\in W\cap {\text {uim}}^-\varphi \cap \Pi _\mathcal {V}(V)$. This implies $W\in \mathcal {V}^-(\varphi )$ and $\Pi _\mathcal {V}(V)\cap W\ne \varnothing$.

By Proposition 6.13 we have

$$\begin{aligned} \varnothing \ne \Pi _\mathcal {V}(V)\cap W\subset (\Pi _\mathcal {V}(V)\setminus V) \cap W \subset \left( \Pi _\mathcal {V}(V)\setminus V\right) \cap \alpha (\varphi ). \end{aligned}$$

Thus, (21) is satisfied.

Now assume that $V\not \in \mathcal {V}^-(\varphi )$.

We have $\Pi _\mathcal {V}(V)={\text {cl}}V \cup V={\text {cl}}V$. Suppose that (21) does not hold. Then

$$\begin{aligned} \varnothing =\left( \Pi _\mathcal {V}(V)\setminus V\right) \cap \alpha (\varphi ) =\left( {\text {cl}}V\setminus V\right) \cap \alpha (\varphi ) ={\text {mo}}V\cap \alpha (\varphi ) \end{aligned}$$

and therefore

$$\begin{aligned} \alpha (\varphi )\setminus V&= \left( {\text {cl}}\alpha (\varphi )\setminus {\text {mo}}\alpha (\varphi )\right) \setminus \left( V\cup {\text {mo}}V\right) \\&= \left( {\text {cl}}\alpha (\varphi )\setminus {\text {mo}}\alpha (\varphi )\right) \setminus {\text {cl}}V = {\text {cl}}\alpha (\varphi )\setminus \left( {\text {mo}}\alpha (\varphi )\cup {\text {cl}}V\right) . \end{aligned}$$

By Proposition 3.10 the set $\alpha (\varphi )\setminus V$ is locally closed as a difference of closed sets. Clearly, $\alpha (\varphi )\setminus V$ is $\mathcal {V}$-compatible. This shows that $\alpha (\varphi )$ is not a minimal locally closed and $\mathcal {V}$-compatible set containing $\bigcup \mathcal {V}^-(\varphi )$. This contradicts Proposition 6.13. Hence (21) holds for $V\subset \alpha (\varphi )$.

The proof of (21) for $V\in \mathcal {V}^+(\varphi )$ is a straightforward adaptation of the proof for $V\subset \alpha (\varphi )$. To see (22) observe that since $\varphi ^{{\text {op}}{}}$ is a full solution of $\mathcal {V}^{{\text {op}}{}}$, $\omega (\varphi ^{{\text {op}}{}})=\alpha (\varphi )$ by Proposition 6.12 and, clearly, $\mathcal {V}^+(\varphi ^{{\text {op}}{}})=\mathcal {V}^-(\varphi )$, we may apply (21) to $\mathcal {V}^{{\text {op}}{}}$, $\varphi ^{{\text {op}}{}}$ and $\omega (\varphi ^{{\text {op}}{}})$. Thus, by Proposition 4.6 we get $\left( \Pi _\mathcal {V}^{-1}(V)\setminus V\right) \cap \alpha (\varphi ) = \left( \Pi _{\mathcal {V}^{{\text {op}}{}}}(V)\setminus V\right) \cap \omega (\varphi ^{{\text {op}}{}})\ne \varnothing ,$ and the claim for $\omega (\varphi )$ follows similarly. $\square$

#### Theorem 6.15

Let $\varphi$ be an essential solution in *X*. Then both limit sets $\alpha (\varphi )$ and $\omega (\varphi )$ are non-empty strongly connected isolated invariant sets.

#### Proof

The nonemptiness of $\alpha (\varphi )$ and $\omega (\varphi )$ follows from Proposition 6.4.

The sets $\alpha (\varphi )$ and $\omega (\varphi )$ are $\mathcal {V}$-compatible and locally closed by Proposition 6.11. In order to prove that they are isolated invariant sets it suffices to apply Proposition 4.13 as long as we prove that $\alpha (\varphi )$ and $\omega (\varphi )$ are also invariant.

We will first prove that $\alpha (\varphi )$ is invariant. Let $x\in \alpha (\varphi )$. Suppose that $\mathcal {V}^-(\varphi )$ is a singleton. Then by Proposition 6.13, $\alpha (\varphi )=[x]_{\mathcal {V}}$. Since $\varphi$ is essential, this is possible only if $[x]_{\mathcal {V}}$ is critical. It follows that the stationary solution $\psi (t)=x$ is essential. Hence $\alpha (\varphi )$ is an isolated invariant set.

Assume now that there are at least two different multivectors in $\mathcal {V}^-(\varphi )$. Then the assumptions of Lemma 6.14 are satisfied and, as a consequence of (21), for every $x\in \alpha (\varphi )$ there exist a point $x'\in [x]_{\mathcal {V}}$ and a $y\in \alpha (\varphi )$ such that $y\in \left( \Pi _\mathcal {V}(x')\setminus [x]_{\mathcal {V}} \right) \cap \alpha (\varphi )$. Hence, we can construct a right-essential solution

$$\begin{aligned} x_0\cdot x'_0\cdot x_1\cdot x'_1\cdot x_2\cdot x'_2\cdot \dotsc , \end{aligned}$$

where $x_0=x$, $x'_i \in [x_i]_{\mathcal {V}}$, and $x_{i+1}\in \left( \Pi _\mathcal {V}(x'_i)\setminus [x_i]_{\mathcal {V}} \right) \cap \alpha (\varphi )$. Property (22) provides a complementary left-essential solution. Concatenation of both solutions gives an essential solution in $\alpha (\varphi )$. Hence, we proved that $\alpha (\varphi )$ is invariant and consequently an isolated invariant set.

Finally, we prove that $\alpha (\varphi )$ is strongly connected. To this end consider points $x,y\in \alpha (\varphi )$. We will show that then ${\text {Path}}_\mathcal {V}(x,y,\alpha (\varphi ))\ne \varnothing$. Using the two abbreviations $V_x:=[x]_{\mathcal {V}}$ and $V_y:=[y]_{\mathcal {V}}$ it is clear that

23 $$\begin{aligned} V_x,V_y\in \mathcal {V}^-(\varphi ) \quad \Rightarrow \quad {\text {Path}}_\mathcal {V}(x,y,\alpha (\varphi ))\ne \varnothing . \end{aligned}$$

Assume now that $V_x\subset \alpha (\varphi )\setminus \bigcup \mathcal {V}^-(\varphi )$ and $V_y\in \mathcal {V}^-(\varphi )$. By (23) it is enough to show that there exists at least one point $z\in \bigcup \mathcal {V}^-(\varphi )$ such that ${\text {Path}}_\mathcal {V}(x, z,\alpha (\varphi ))\ne \varnothing$. Suppose the contrary.

The set $\pi _\mathcal {V}^+(V_x,\alpha (\varphi ))$ is closed and $\mathcal {V}$-compatible in $\alpha (\varphi )$ by Proposition 5.11. By Proposition 3.5 set $A:=\alpha (\varphi )\setminus \pi _\mathcal {V}^+(V_x,\alpha (\varphi ))$ is locally closed. Clearly, it is $\mathcal {V}$-compatible and contains $\bigcup \mathcal {V}^-(\varphi )$. Yet this results in a contradiction, because we have now found a smaller $\mathcal {V}$-hull for $\bigcup \mathcal {V}^-(\varphi )$.

Now, consider the case when $V_x\in \mathcal {V}^-(\varphi )$ and $V_y\subset \alpha (\varphi )\setminus \bigcup \mathcal {V}^-(\varphi )$. The set $\pi _\mathcal {V}^+(V_x,\alpha (\varphi ))$ is $\mathcal {V}$-compatible and locally closed by Proposition 5.11. In view of (23) we have $\bigcup \mathcal {V}^-(\varphi )\subset \pi _\mathcal {V}^+(V_x,\alpha (\varphi ))$. Thus, one either has $V_y\subset \pi _\mathcal {V}^+(V_x,\alpha (\varphi ))$ or $\pi _\mathcal {V}^+(V_x,\alpha (\varphi ))$ is a smaller $\mathcal {V}$-compatible, locally closed set containing $\bigcup \mathcal {V}^-(\varphi )$. In both cases we get a contradiction.

Finally, let $V_x,V_y\subset \alpha (\varphi )\setminus \bigcup \mathcal {V}^-(\varphi )$ and let $z\in \bigcup \mathcal {V}^-(\varphi )$. Using the previous cases we can find $\psi _1\in {\text {Path}}_\mathcal {V}(x,z,\alpha (\varphi ))$ and $\psi _2\in {\text {Path}}_\mathcal {V}(z,y,\alpha (\varphi ))$. Then, $\psi _1\cdot \psi _2\in {\text {Path}}_\mathcal {V}(x,y,\alpha (\varphi ))$. This finishes the proof that $\alpha (\varphi )$ is strongly connected.

The proof for $\omega (\varphi )$ is analogous. $\square$

Let $\varphi$ be an essential solution. We say that an isolated invariant set *S* *absorbs* $\varphi$ *in positive* (respectively negative) *time* if $\varphi (t)\in S$ for all $t\ge t_0$ (for all $t\le t_0$) for some $t_0\in {\mathbb {Z}}$. We denote by $\Omega (\varphi )$ (respectively by $\mathcal {A}(\varphi )$) the family of isolated invariant sets absorbing $\varphi$ in positive (respectively negative) time.

#### Proposition 6.16

For an essential solution $\varphi$ we have

24 $$\begin{aligned} \alpha (\varphi )&=\bigcap \mathcal {A}(\varphi ), \end{aligned}$$

25 $$\begin{aligned} \omega (\varphi )&=\bigcap \Omega (\varphi ). \end{aligned}$$

#### Proof

Let $\varphi$ be an essential solution. It follows from Proposition 6.4 that there exists a $k\in {\mathbb {Z}}^-$ such that $\varphi ((-\infty ,k])={\text {uim}}^-\varphi \subset \alpha (\varphi )$. Moreover, by Proposition 6.15 we have $\alpha (\varphi )\in \mathcal {A}(\varphi )$. Hence $\bigcap \mathcal {A}(\varphi )\subset \alpha (\varphi )$. To see the opposite inclusion take an $S\in \mathcal {A}(\varphi )$. Then, there exists a $t_0\in {\mathbb {Z}}^-$ such that $\varphi ((-\infty ,t_0])\subset S$. It follows that

$$\begin{aligned} \alpha (\varphi )=\langle {\text {uim}}^-\varphi \rangle _\mathcal {V}\subset \langle \varphi ((-\infty , t_0])\rangle _\mathcal {V}\subset \langle S\rangle _\mathcal {V}=S. \end{aligned}$$

Hence, $\alpha (\varphi )\subset \mathcal {A}(\varphi )$. This proves (24). The proof of (25) is analogous. $\square$

Let $A,B\subset X$. We define the *connection set from* *A* *to* *B* by:

26 $$\begin{aligned} C(A,B) := \left\{ x\in X\ |\ \exists _{\varphi \in {\text {eSol}}(x,X)}\ \alpha (\varphi )\subset A\ \text {and}\ \omega (\varphi )\subset B \right\} . \end{aligned}$$

#### Proposition 6.17

Assume $A,B\subset X$. Then the connection set *C*(*A*, *B*) is an isolated invariant set.

#### Proof

To prove that *C*(*A*, *B*) is invariant, take an $x\in C(A,B)$ and choose a $\varphi \in {\text {eSol}}(x,X)$ as in (26). It is clear that $\varphi (t)\in C(A,B)$ for every $t\in {\mathbb {Z}}$. Thus, $\varphi \in {\text {eSol}}(x,C(A,B))$, and this in turn implies $x\in {\text {Inv}}C(A,B)$ and shows that *C*(*A*, *B*) is invariant. Now consider a point $y\in [x]_{\mathcal {V}}$. Then the solution $\rho =\varphi ^-\cdot y\cdot \varphi ^+$ is a well-defined essential solution through *y* such that $\alpha (\rho )\subset A$ and $\omega (\rho )\subset B$. Thus, *C*(*A*, *B*) is $\mathcal {V}$-compatible.

In order to prove that *C*(*A*, *B*) is locally closed, consider $x,z\in C(A,B)$, and a $y\in X$ such that $z\le _\mathcal {T}y\le _\mathcal {T}x$. Select essential solutions $\varphi _x\in {\text {eSol}}(x,C(A,B))$ and $\varphi _z\in {\text {eSol}}(z,C(A,B))$. Then $\psi :=\varphi ^-_x\cdot y\cdot \varphi ^+_z$ is a well-defined essential solution through *y* such that $\alpha (\psi )\subset A$ and $\omega (\psi )\subset B$. It follows that $y\in C(A,B)$. Thus, by Proposition 3.10, *C*(*A*, *B*) is locally closed. Finally, Proposition 4.13 proves that *C*(*A*, *B*) is an isolated invariant set. $\square$

#### Proposition 6.18

Assume *A* is an attractor. Then $C(A, A^\star )=\varnothing$. Similarly, if *R* is a repeller, then $C(R^\star ,R)=\varnothing$.

#### Proof

Suppose there exists an $x\in C(A, A^\star )$. Then by (26) we can choose a $\varphi \in {\text {eSol}}(x,X)$ and a $t\in {\mathbb {Z}}$ such that $\varphi (t)\in A$ and $\varphi (t+1)\not \in A$. However, since *A* is an attractor, $\varphi (t)\in A$ implies $\varphi (t+1)\in A$, a contradiction. The proof for a repeller is analogous. $\square$

## Morse decomposition, Morse equation, Morse inequalities

In this final section we define Morse decompositions and prove the Morse inequalities for combinatorial multivector fields. We recall the general assumption that *X* is invariant with respect to a fixed combinatorial multivector field $\mathcal {V}$ on *X*.

### Morse decompositions

#### Definition 7.1

Assume *X* is invariant and $({\mathbb {P}},\le )$ is a finite poset. Then the collection $\mathcal {M}={\{\,M_p\mid p\in {\mathbb {P}}\,\}}$ is called a *Morse decomposition of* *X* if the following conditions are satisfied:

(i) $\mathcal {M}$ is a family of mutually disjoint, isolated invariant subsets of *X*.
(ii) For every essential solution $\varphi$ in *X* either ${\text {im}}{\varphi }\subset M_r$ for an $r\in {\mathbb {P}}$ or there exist $p,q\in {\mathbb {P}}$ such that $q > p$ and
  $$\begin{aligned} \alpha (\varphi )\subset M_q\quad \text {and}\quad \omega (\varphi )\subset M_p. \end{aligned}$$

We refer to the elements of $\mathcal {M}$ as *Morse sets*.

**Fig. 11:**
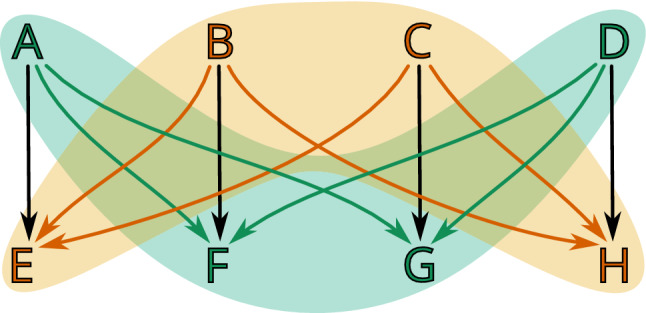
A sample combinatorial multivector field $\mathcal {V}=\left\{ \{A,D,F,G\}, \{B,C,E,H\}\right\}$ on the finite topological space $X=\left\{ A, B,C,D,E,F,G,H\right\}$ with Alexandroff topology induced by the partial order indicated by arrows. If we consider $\mathcal {M}=\mathcal {V}$, then one obtains a partition into isolated invariant sets with $X\setminus \mathcal {M}=\varnothing$. Note that $\dots D\cdot H\cdot B\cdot F\cdot D\cdot \dots$ is a periodic trajectory which passes through both “Morse sets”

Note that in the classical definition of Morse decomposition the analogue of condition (ii) is formulated in terms of trajectories passing through points $x \not \in \bigcup \mathcal {M}$. In our setting we have to consider all possible solutions. There are two reasons for that: the non-uniqueness of a solution passing through a point and the tightness of finite topological spaces. In particular, in the finite topological space setting it is possible to have a non-trivial Morse decomposition such that every point is contained in a Morse set. Without our modification of the definition of Morse decomposition, recurrent behavior spreading into several sets is a distinct possibility. Figure 11 illustrates such an example.

#### Proposition 7.2

Let *X* be an invariant set, let $A\subset X$ be an attractor, and let $A^\star$ denote its non-empty dual repeller. Furthermore, define $M_1:=A$, $M_2:=A^\star$, and let ${\mathbb {P}}:=\{1,2\}$ be an indexing set with the order induced from ${\mathbb {N}}$. Then $\mathcal {M}=\{M_1, M_2\}$ is a Morse decomposition of *X*.

#### Proof

By Theorems 6.2 and 6.3 both *A* and $A^\star$ are isolated invariant sets which are clearly disjoint. Let $x\in X$ and let $\varphi \in {\text {eSol}}_\mathcal {V}(x,X)$. By Theorem 6.15 the set $\omega (\varphi )$ is strongly connected and invariant. It is also indecomposable by Proposition 6.7. By Proposition 6.9 it is either a subset of *A* or a subset of ${\text {Inv}}(X\setminus A)=A^\star$. The same holds for $\alpha (\varphi )$.

We therefore have four cases. The situation $\alpha (\varphi )\subset M_2$ and $\omega (\varphi )\subset M_1$ is consistent with the definition. The case $\alpha (\varphi )\subset M_1$ and $\omega (\varphi )\subset M_2$ is clearly in conflict with the definition of an attractor and a repeller. Now suppose that we have $\alpha (\varphi )\subset M_1$ and $\omega (\varphi )\subset M_1$. It follows that there exists a $t\in {\mathbb {Z}}$ such that $\varphi ((-\infty ,t]))\subset A$. Since *A* is an attractor we therefore have $\varphi (t+1)\in \Pi _\mathcal {V}(\varphi (t)) \subset A$, and induction easily implies ${\text {im}}\varphi \subset A=M_1$. The same argument holds for $M_2$. $\square$

### Strongly connected components as Morse decomposition

We recall that $G_\mathcal {V}$ stands for the digraph interpretation of the multivalued map $\Pi _\mathcal {V}$ associated with the multivector field $\mathcal {V}$ on *X*.

#### Theorem 7.3

Assume *X* is invariant. Consider the family $\mathcal {M}$ of all strongly connected components *M* of $G_\mathcal {V}$ with ${\text {eSol}}(M)\ne \varnothing$. Then $\mathcal {M}$ is a minimal Morse decomposition of *X*.

#### Proof

For convenience, assume that $\mathcal {M}=\{M_i\mid i\in {\mathbb {P}}\}$ is bijectively indexed by a finite set ${\mathbb {P}}$. Any two strongly connected components $M_i,M_j\in \mathcal {M}$ are clearly disjoint and by Theorem 4.16 they are isolated invariant sets. Hence, condition (i) of a Morse decomposition is satisfied.

Define a relation $\le$ on the indexing set ${\mathbb {P}}$ by

$$\begin{aligned} i\le j\ \Leftrightarrow \ \exists _{\varphi \in {\text {Path}}_\mathcal {V}(X)}\ \varphi ^{\sqsubset }\in M_j\ \text {and}\ \varphi ^{\sqsupset }\in M_i. \end{aligned}$$

It is clear that $\le$ is reflexive. To see that it is transitive consider $M_i,M_j,M_k\in \mathcal {M}$ such that $k\le j\le i$. It follows that there exist paths $\varphi$ and $\psi$ such that $\varphi ^{\sqsubset }\in M_i$, $\varphi ^{\sqsupset }, \psi ^{\sqsubset }\in M_j$ and $\psi ^{\sqsupset }\in M_k$. Since $M_j$ is strongly connected we can find $\rho \in {\text {Path}}_\mathcal {V}(\varphi ^{\sqsupset },\psi ^{\sqsubset },X)$. The path $\varphi \cdot \rho \cdot \psi$ clearly connects $M_i$ with $M_k$ proving that $k\le i$.

In order to show that $\le$ is antisymmetric consider sets $M_i,M_j$ with $i\le j$ and $j\le i$. It follows that there exist paths $\varphi$ and $\psi$ such that $\varphi ^{\sqsubset },\psi ^{\sqsupset }\in M_i$ and $\varphi ^{\sqsupset },\psi ^{\sqsubset }\in M_j$. Since the sets $M_i,M_j$ are strongly connected we can find paths $\rho$ and $\rho '$ from $\varphi ^{\sqsupset }$ to $\psi ^{\sqsubset }$ and from $\psi ^{\sqsupset }$ to $\varphi ^{\sqsubset }$ respectively. Clearly, $\varphi \in {\text {Path}}_\mathcal {V}(\varphi ^{\sqsubset },\varphi ^{\sqsupset }, X)$ and $\rho \cdot \psi \cdot \rho '\in (\varphi ^{\sqsupset },\varphi ^{\sqsubset }, X)$. This proves that $M_i$ and $M_j$ are the same strongly connected component.

Let $x\in X$ and let $\varphi \in {\text {eSol}}(x,X)$. We will prove that $\alpha (\varphi )\subset M_q$ and $\omega (\varphi )\subset M_p$ for some $M_p,M_q\in \mathcal {M}$. Note that $\varphi ^{-1}(V)$ is right-infinite for any $V\in \mathcal {V}^+(\varphi )$. It follows that for any $V, W\in \mathcal {V}^+(\varphi )$ we can find $0<t_0<t_1<t_2$ such that $\varphi (t_0),\varphi (t_2)\in V$ and $\varphi (t_1)\in W$. Thus, points in $\bigcup \mathcal {V}^+(\varphi )$ are in the same strongly connected component and therefore $\bigcup \omega _\mathcal {V}(\varphi )\subset C$ for some strongly path connected component of $G_\mathcal {V}$. Moreover, $\dotsc \cdot \varphi |_{[t_0,t_2]}\cdot \varphi |_{[t_0,t_2]}\cdot \dotsc$ is clearly an essential solution in *C*. Thus $C=M_p\in \mathcal {M}$ for some $p\in {\mathbb {P}}$. By Proposition 4.15 the set $M_p$ is $\mathcal {V}$-compatible and locally closed. Hence, $M_p$ is a superset of the $\mathcal {V}$-hull of $\bigcup \mathcal {V}^+(\varphi )$ and from Proposition 6.13 we get $\omega (\varphi )\subset M_p$. A similar argument gives $\alpha (\varphi )\subset M_q$ for some $q\in {\mathbb {P}}$. It is clear from the definition of $\le$ that $p\le q$.

Next, we show that $\alpha (\varphi )\subset M\in \mathcal {M}$ and $\omega (\varphi )\subset M$ implies ${\text {im}}\varphi \subset M$. Thus, take a $y\in {\text {im}}\varphi$. Then $y=\varphi (t_1)$ for some $t_1\in {\mathbb {Z}}$. Since $\alpha (\varphi )$ and $\omega (\varphi )$ are subsets of *M*, we can find $t_0<t_1$ and $t_2>t_1$, such that $x:=\varphi (t_0)\in M$ and $z:=\varphi (t_2)\in M$. Since *M* is strongly connected there exists a path $\rho$ from *z* to *x*. Then $\varphi |_{[t_1,t_2]} \cdot \rho \in {\text {Path}}_\mathcal {V}(y,x,X)$. Since $\varphi |_{[t_0,t_1]}\in {\text {Path}}_\mathcal {V}(x,y,X)$, we conclude that *y* belongs to the strongly connected component of *x*, that is, $y\in M$. This completes the proof that $\mathcal {M}$ is a Morse decomposition.

To show that $\mathcal {M}$ is a minimal Morse decomposition assume the contrary. Then we can find a Morse decomposition $\mathcal {M}'$ of an $M\in \mathcal {M}$ with at least two different Morse sets $M_1$ and $M_2$ in $\mathcal {M}'$. Since $M_1$ and $M_2$ are disjoint and $\mathcal {V}$-compatible we can find disjoint multivectors $V_1\subset M_1$ and $V_2\subset M_2$. Since the set *M* is strongly connected we can find paths $\varphi \in {\text {Path}}_\mathcal {V}(x,y,M)$ and $\rho \in {\text {Path}}_\mathcal {V}(y,x,M)$ with $x\in V_1$ and $y\in V_2$. The alternating concatenation of these paths $\psi :=\dotsc \cdot \varphi \cdot \rho \cdot \varphi \cdot \rho \dotsc$ is a well-defined essential solution. Then $\varnothing \ne {\text {im}}\psi \subset \alpha (\psi )\cap \omega (\psi )$ which implies ${\text {im}}\psi \subset M_3$ for an $M_3\in \mathcal {M}'$. However, ${\text {im}}\psi \cap M_1\ne \varnothing \ne {\text {im}}\psi \cap M_2$, a contradiction. $\square$

### Morse sets

For a subset $I\subset {\mathbb {P}}$ we define the *Morse set of* *I* by

$$\begin{aligned} M(I):=\bigcup _{i,j\in I} C(M_i,M_j). \end{aligned}$$

#### Theorem 7.4

The set *M*(*I*) is an isolated invariant set.

#### Proof

Observe that *M*(*I*) is invariant, because, by Proposition 6.17, every connection set is invariant, and by Proposition 4.7 the union of invariant sets is invariant. We will prove that *M*(*I*) is locally closed. To see that, suppose the contrary. Then, by Proposition 3.10, we can choose $a,c\in M(I)$ and a point $b\not \in M(I)$ such that $c\le _\mathcal {T}b\le _\mathcal {T}a$. There exist essential solutions $\varphi _a\in {\text {eSol}}(a,X)$ and $\varphi _c\in {\text {eSol}}(c,X)$ such that $\alpha (\varphi _a)\subset M_q$ and $\omega (\varphi _c)\subset M_p$ for some $p,q\in I$. It follows that $\psi :=\varphi _a^-\cdot b\cdot \varphi _c^+$ is a well-defined essential solution such that $\alpha (\psi )\subset M_q$ and $\omega (\psi )\subset M_p$. Hence, $b\in C(M_q,M_p)\subset M(I)$ which proves that *M*(*I*) is locally closed. Moreover, *M*(*I*) is $\mathcal {V}$-compatible as a union of $\mathcal {V}$-compatible sets. Thus, the conclusion follows from Proposition 4.13. $\square$

#### Theorem 7.5

If *I* is a down set in ${\mathbb {P}}$, then *M*(*I*) is an attractor in *X*.

#### Proof

We will show that *M*(*I*) is closed. For this, let $x\in {\text {cl}}(M(I))$. By Proposition 3.7 we can choose a $y\in M(I)$ such that $x\in {\text {cl}}y$. Consider essential solutions $\varphi _x\in {\text {eSol}}(x,X)$ and $\varphi _y\in {\text {eSol}}(y,M(I))$ with $\alpha (\varphi _y)\subset M_i$ for some $i\in I$. The concatenated solution $\varphi :=\varphi _y^-\cdot \varphi _x^+$ is well-defined and satisfies $\alpha (\varphi )\subset M_i$ and $\omega (\varphi )\subset M_j$ for some $j\in {\mathbb {P}}$. Definition 7.1 implies that $i>j$. Since *I* is a down set, we get $j\in I$. It follows that $x\in C(M_i,M_j)\subset M(I)$. Thus, ${\text {cl}}M(I)\subset M(I)$, which proves that *M*(*I*) is closed. Finally, Theorem 6.2 implies that the set *M*(*I*) is an attractor. $\square$

#### Theorem 7.6

If $I\subset {\mathbb {P}}$ is convex, then $(M(I^\le ), M(I^<))$ is an index pair for the isolated invariant set *M*(*I*).

#### Proof

By Proposition 3.1 the sets $I^\le$ and $I^<$ are down sets. Thus, by Theorem 7.5 both $M(I^\le )$ and $M(I^<)$ are attractors. It follows that $\Pi _\mathcal {V}(M(I^\le ))\subset M(I^\le )$ and $\Pi _\mathcal {V}(M(I^<))\subset M(I^<)$. Therefore, conditions (IP1) and (IP2) of an index pair are satisfied.

Let $A:= M(I^\le )\setminus M(I^<)$. The set *A* is $\mathcal {V}$-compatible as a difference of $\mathcal {V}$-compatible sets. By Proposition 3.3 it is also locally closed, because $M(I^\le )$ and $M(I^<)$ are closed as attractors (see Theorems 6.2 and 6.3). We claim that $M(I)\subset A$. To see this, assume the contrary and select an $x\in M(I)\setminus A$. By the definition of *M*(*I*) we can find an essential solution $\varphi$ through *x* such that $\omega (\varphi )\subset M_p$ for some $p\in I$. Since $M(I)\subset M(I^\le )$ and $x\not \in A$ we get $x\in M(I^<)$. But $M(I^<)$ is an attractor. Therefore $\omega (\varphi )\subset M(I^<)$, which in turn implies $p\not \in I$, a contradiction.

To prove the opposite inclusion take an $x\in {\text {Inv}}(M(I^\le )\setminus M(I^<))$. Then we can find an essential solution $\varphi \in {\text {eSol}}(x,M(I^\le )\setminus M(I^<))$, and clearly one has ${\text {im}}\varphi \subset M(I^\le )\setminus M(I^<)$. In particular,

27 $$\begin{aligned} {\text {uim}}^-\varphi \cap M(I^<)=\varnothing \quad \text {and}\quad {\text {uim}}^+\varphi \cap M(I^<)=\varnothing . \end{aligned}$$

We also have $\varphi \in {\text {eSol}}(x, M(I^\le ))$, which means that there exist $p,q\in I^\le$ such that $p\ge q$, $\alpha (\varphi )\subset M_p$, $\omega (\varphi )\subset M_q$. We cannot have $p\in I^<$, because then we get $\varnothing \ne {\text {uim}}^-\varphi \subset \alpha (\varphi )\subset M_p\subset M(I^<)$ which contradicts (27). Therefore, $p\in I^\le \setminus I^<=I$. By an analogous argument we get $q\in I$. It follows that $x\in C(M_p,M_q)\subset M(I)$. $\square$

Since for a down set $I\subset {\mathbb {P}}$ we have $I^\le =I$, $I^<=\varnothing$, as an immediate consequence of Theorem 7.6 we get the following corollary.

#### Corollary 7.7

If *I* is a down set in ${\mathbb {P}}$, then $I^\le = I$, $I^<=\varnothing$, $(M(I),\varnothing )$ is an index pair for *M*(*I*).

#### Theorem 7.8

Assume *X* is invariant, $A\subset X$ is an attractor and $A^\star$ is its dual repeller. Then we have

28 $$\begin{aligned} p_A(t) + p_{A^\star }(t) = p_X(t) + (1+t)q(t) \end{aligned}$$

for a polynomial *q*(*t*) with non-negative coefficients. Moreover, if $q\ne 0$, then $C(A^\star , A)\ne \varnothing$.

#### Proof

Let ${\mathbb {P}}:=\{1,2\}$ with order induced from ${\mathbb {N}}$, $M_1:=A$ and $M_2:=A^\star$. Then $\mathcal {M}:=\{M_1,M_2\}$ is a Morse decomposition of *X* by Proposition 7.2. For $I:=\{2\}$ one obtains $I^\le =\{1,2\}$ and $I^<=\{1\}$. Yet, this immediately implies both $M(I^\le )=X$ and $M(I^<)=M(\{1\})=A$.

We have

29 $$\begin{aligned} p_X(t)=p_{M(I^\le )}(t) \quad \text{ and }\quad p_A(t)=p_{M(I^<)}(t). \end{aligned}$$

By Theorem 7.6 the pair $(M(I^\le ), M(I^<))$ is an index pair for $M(I)=A^\star$. Thus, by substituting $P_1:=M(I^\le )$, $P_2:=M(I^<)$, $S:=A^\star$ into (14) in Corollary 5.17 we get (28) from (29). By Proposition 6.18 we have the identity $C(A,A^\star )=\varnothing$. Therefore, if in addition $C(A^\star , A)=\varnothing$, then *X* decomposes into *A* and $A^\star$, and Theorem 5.19 implies

$$\begin{aligned} H(P_1)={\text {Con}}(X)={\text {Con}}(A)\oplus {\text {Con}}(A^\star )=H(P_2)\oplus H(A^\star ), \end{aligned}$$

as well as $q=0$ in view of Proposition 5.17. This finally shows that $q\ne 0$ implies $C(A^\star , A)\ne \varnothing$. $\square$

### Morse equation and Morse inequalities

The following two theorems follow from the results of the preceding section by adapting the proofs of the corresponding results in Mrozek (2017).

#### Theorem 7.9

Assume *X* is invariant and ${\mathbb {P}}=\{1,2,...,n\}$ is ordered by the linear order of the natural numbers. Let $\mathcal {M}:=\{M_p\ |\ p\in {\mathbb {P}}\}$ be a Morse decomposition of *X* and set $A_i:= M(\{i\}^{\le })$, $A_0:=\varnothing$. Then $(A_{i-1},M_i)$ is an attractor-repeller pair in $A_i$. Moreover,

$$\begin{aligned} \sum _{i=1}^{n} p_{M_i}(t) = p_X(t) + (1+t)\sum _{i=1}^n q_i(t) \end{aligned}$$

for some polynomials $q_i(t)$ with non-negative coefficients and such that $q_i(t)\ne 0$ implies $C(M_i,A_{i-1})\ne \varnothing$ for $i=2,3,...,n$.

As before, for a locally closed set $A\subset X$ we define its *k*th Betti number by $\beta _k(A):={\text {rank}}H_k({\text {cl}}A, {\text {mo}}A)$.

#### Theorem 7.10

Assume *X* is invariant. For a Morse decomposition $\mathcal {M}$ of *X* define

$$\begin{aligned} m_k(\mathcal {M}):=\sum _{r\in {\mathbb {P}}}\beta _k(M_r). \end{aligned}$$

Then for any $k\in {\mathbb {Z}}^+$ we have the following inequalities.

(i) The strong Morse inequalities:
  $$\begin{aligned} m_k(\mathcal {M}) - m_{k-1}(\mathcal {M}) + ... \pm m_0(\mathcal {M}) \ge \beta _k(X) - \beta _{k-1}(X) + ... \pm \beta _0(X), \end{aligned}$$
(ii) The weak Morse inequalities:
  $$\begin{aligned} m_k(\mathcal {M}) \ge \beta _k(X). \end{aligned}$$
